# Hepatic T-cell senescence and exhaustion are implicated in the progression of fatty liver disease in patients with type 2 diabetes and mouse model with nonalcoholic steatohepatitis

**DOI:** 10.1038/s41419-023-06146-8

**Published:** 2023-09-21

**Authors:** Byeong Chang Sim, Yea Eun Kang, Sun Kyoung You, Seong Eun Lee, Ha Thi Nga, Ho Yeop Lee, Thi Linh Nguyen, Ji Sun Moon, Jingwen Tian, Hyo Ju Jang, Jeong Eun Lee, Hyon-Seung Yi

**Affiliations:** 1 Laboratory of Endocrinology and Immune System, Chungnam National University School of Medicine, Daejeon, Republic of Korea; 2 Department of Medical Science, Chungnam National University School of Medicine, Daejeon, Republic of Korea; 3 Department of Internal Medicine, Chungnam National University School of Medicine, Daejeon, Republic of Korea; 4 Department of Radiology, Chungnam National University Hospital, Daejeon, Republic of Korea

**Keywords:** Translational research, Non-alcoholic fatty liver disease

## Abstract

Immunosenescence and exhaustion are involved in the development and progression of type 2 diabetes (T2D) and metabolic liver diseases, including fatty liver, fibrosis, and cirrhosis, in humans. However, the relationships of the senescence and exhaustion of T cells with insulin resistance-associated liver diseases remain incompletely understood. To better define the relationship of T2D with nonalcoholic fatty liver disease, 59 patients (mean age 58.7 ± 11.0 years; 47.5% male) with T2D were studied. To characterize their systemic immunophenotypes, peripheral blood mononuclear cells were analyzed using flow cytometry. Magnetic resonance imaging (MRI)-based proton density fat fraction and MRI-based elastography were performed using an open-bore, vertical-field 3.0 T scanner to quantify liver fat and fibrosis, respectively. The participants with insulin resistance had a significantly larger population of CD28 − CD57+ senescent T cells among the CD4+ and CD8 + T cells than those with lower Homeostatic Model Assessment for Insulin Resistance (HOMA-IR) values. The abundances of senescent CD4+ and CD8 + T cells and the HOMA-IR positively correlated with the severity of liver fibrosis, assessed using MRI-based elastography. Interleukin 15 from hepatic monocytes was found to be an inducer of bystander activation of T cells, which is associated with progression of liver disease in the participants with T2D. Furthermore, high expression of genes related to senescence and exhaustion was identified in CD4+ and CD8 + T cells from the participants with nonalcoholic steatohepatitis or liver cirrhosis. Finally, we have also demonstrated that hepatic T-cell senescence and exhaustion are induced in a diet or chemical-induced mouse model with nonalcoholic steatohepatitis. In conclusion, we have shown that T-cell senescence is associated with insulin resistance and metabolic liver disease in patients with T2D.

## Introduction

The liver consists of parenchymal cells (hepatocytes) and nonparenchymal cells, including lymphocytes, which are found scattered not only around the portal tracts and the perivenular areas but also throughout the hepatic parenchyma. A typical adult human liver contains approximately 10^9^–10^10^ innate and adaptive lymphocytes [1]. T-cell extravasation and trafficking into the parenchyma are facilitated by small pores in the sinusoidal lining of endothelial cells [2]. In addition, portal blood, which contains a wide variety of bacterial and environmental pathogens, flows directly to the liver, bypassing classical immune sentinel tissues such as the spleen and lymph nodes [3]. However, the liver can mediate local and systemic tolerance to self and foreign antigens through the effects of specialized resident immune cells that produce anti-inflammatory cytokines and ligands that inhibit T-cell activation and proliferation [4]. This hepatic immune homeostasis requires a combination of unique hepatic architecture, the infiltration of and residency by immune cells, and immune surveillance. Perturbation of this balance initiates and promotes persistent liver injury and inflammation, resulting in liver pathology such as nonalcoholic steatohepatitis (NASH), liver fibrosis, and cirrhosis [5].

T-cell activation and differentiation are regulated in the context in which naïve T cells encounter antigen, but this can result in T-cell dysfunction, including exhaustion or senescence, as well as defects in effector-mediated immunity, which is important for host defense [6]. The senescence and exhaustion of immune cells are key instigators of chronic inflammation as part of the pathogenesis of metabolic diseases. Senescent T-cell-mediated inflammatory responses are associated with the pathogenesis of acute coronary syndrome and hypertension [7, 8], and T-cell senescence is also linked to obesity and type 2 diabetes (T2D) in humans and mice [9, 10]. This crosstalk between immunosenescence and metabolic diseases may be mediated by greater secretion of proinflammatory cytokines, reactive oxygen species, and acute-phase reactants by senescent immune cells. Moreover, T-cell exhaustion, in which dysfunctional T cells produce smaller quantities of cytokines and show impaired immune surveillance, is a feature of pathologies ranging from simple metabolic liver disease to NASH-associated hepatocellular carcinoma [11]. CD8 + T cells also exhibit dysfunction induced by chronic antigenic stimulation during viral infections, cirrhosis, and tumor development [6, 12]. However, the relationships of the senescence and exhaustion of T cells with liver disease, ranging from simple steatosis to cirrhosis, remain to be fully elucidated.

The progression of nonalcoholic fatty liver disease (NAFLD) is closely associated with insulin resistance-induced hyperglycemia and involves a combination of oxidative stress and inflammation [13, 14]. Hepatic insulin resistance leads to uncontrolled gluconeogenesis, glycogenolysis, lipogenesis, and dyslipidemia, resulting in diabetic liver complications such as NASH, liver fibrosis, cirrhosis, and hepatocellular carcinoma [13]. However, it is unclear whether insulin resistance or T2D-associated immunosenescence accelerates the progression of metabolic liver disease. Therefore, in the present study, we aimed to evaluate liver fat and fibrosis using magnetic resonance imaging (MRI) and ultrasonography, and to characterize the composition of the peripheral blood mononuclear cells (PBMCs) of patients with T2D. We further aimed to characterize the relationships of metabolic liver diseases with the immunosenescence or exhaustion of hepatic immune cells in humans through unbiased single-cell transcriptomic profiling.

## Materials and methods

### Design, study sample, and setting

Of the 88 participants initially enrolled in 2021, 20 had viral hepatitis or cirrhosis, consumed a substantial amount of alcohol (men: >40 g/day, women: >20 g/day), or had chronic inflammatory disease at baseline, and were therefore excluded. We also excluded nine participants who had been administering glucagon-like peptide 1 receptor agonists, sodium-glucose cotransporter 2 inhibitors, or peroxisome proliferator-activated receptor gamma agonists, which might have affected their hepatic inflammation and fibrosis. Thus, data from 59 participants were analyzed. All T2D participants were diagnosed with measurement of serum HbA1c levels in the Chungnam National University Hospital. The study was performed in accordance with the principles of the Declaration of Helsinki and was approved by the Institutional Review Board of the Chungnam National University Hospital (CNUH 2019-04-027). Written consent, documented by the Department of Radiology of Chungnam National University in South Korea, was obtained from all the participants prior to their inclusion in the study.

### Blood sampling and serum biochemistry

Peripheral blood samples were collected after overnight fasting (10–12 h) for the measurement of glucose, insulin, and C-peptide concentrations. Plasma glucose concentration was measured using an automated biochemistry analyzer (RX Daytona, Randox, Crumlin, UK). Plasma insulin was measured using an immunoradiometric assay kit (DIAsource INS-IRMA kit, DIAsource, Louvain-la-Neuve, Belgium). C-peptide concentrations were measured using an Immulite 1000 (Siemens, Munich, Germany). The lipid profile of the participants (low-density lipoprotein-cholesterol, high-density lipoprotein-cholesterol, total cholesterol, and triglyceride concentrations) was evaluated using a blood chemistry analyzer (Hitachi 47; Hitachi, Tokyo, Japan). Glycosylated hemoglobin was quantified using high-performance liquid chromatography (BioRad, Hercules, CA, USA). The aspartate transaminase and alanine transaminase activities were measured using the International Federation of Clinical Chemistry Ultraviolet methods without pyridoxal phosphate (TBA-2000FR; Toshiba, Tokyo, Japan). High-sensitivity C-reactive protein was measured using the photometric latex agglutination method (TBA-2000FR; Toshiba). Homeostatic model assessment-insulin resistance (HOMA-IR) was calculated as fasting serum insulin (µU/mL) × fasting plasma glucose (mg/dL) / 405. HOMA-β was calculated as 360 × fasting insulin (µU/mL) / (fasting plasma glucose (mg/dL) – 63). Adiponectin levels were measured in the sera of the participants with T2D by human adiponectin ELISA kit (DRG International, Inc. USA) with a sensitivity of 0.6 ng/ml.

### Preparation of peripheral blood mononuclear cells for flow cytometry

Peripheral blood samples (10 mL each) were obtained from all the participants diagnosed with T2D and were transferred aseptically to 50 mL polystyrene centrifuge tubes containing EDTA (Sigma-Aldrich, St. Louis, MO, USA) as an anticoagulant and gently mixed. PBMCs were then isolated using Ficoll-Paque density-gradient (GE Healthcare Life Science, Chicago, IL, USA) centrifugation at room temperature. After centrifugation, the PBMC layer was collected and washed with Dulbecco’s PBS. The isolated PBMCs were resuspended in 2 mL Rosewell Park Memorial Institute (RPMI)-1640 medium (Welgene, Daegu, South Korea), and trypan blue dye exclusion testing was used to determine the number of viable cells in the suspension. The isolated PBMCs were cryopreserved in liquid nitrogen until flow cytometric analysis.

### Isolation of human hepatic mononuclear cells

Liver samples from the study participants were cut into small ( < 2 mm) pieces and incubated in prewarmed media containing dissociation enzymes (Miltenyi Biotec, Bergisch Gladbach, Germany) for 30 min at 37 °C. After enzymatic digestion, the hepatic cell suspensions were rapidly homogenized in C-tubes using a GentleMACS Dissociator (Miltenyi Biotec) and the m_liver_03 program. After debris removal, the cells were resuspended in PBS and centrifuged at 1000 × *g* for 5 min for the removal of hepatocytes. The supernatants were filtered through a cell strainer with a 70 μm nylon filter (BD Falcon, Millville, NJ, USA). Hepatic mononuclear cells were isolated by centrifugation at 1200 × *g* for 10 min at 4 °C and resuspended in RPMI-1640 medium (Welgene). The numbers of viable isolated liver mononuclear cells were then quantified, and the cells were used for analysis of the population and function of tissue-resident immune cells using flow cytometry staining (FACS) buffer.

### Isolation of hepatic mononuclear cells in mice

Liver mononuclear cells were isolated, as previously described. Liver tissues from mice were cut into small pieces and incubated with prewarmed media that included dissociation enzymes (Miltenyi Biotec, Bergisch Gladbach, Germany) for 30 min at 37 °C. After enzymatic digestion, the hepatic cell suspensions were quickly homogenized in C-Tubes using the GentleMACS Dissociator (Miltenyi Biotec) and the m_liver_03 program. After debris removal, the cells were resuspended in phosphate-buffered saline and centrifuged at 1000 × *g* for 5 min for hepatocyte elimination. The supernatants were removed by mechanical suction and filtered through a cell strainer with a 70 μm nylon filter (BD Falcon, Millville, NJ). Hepatic mononuclear cells were isolated by centrifugation at 1200 × *g* for 10 min at 4 °C and resuspended in RPMI-1640 medium (Welgene, Daegu, South Korea). The cells underwent single-cell transcriptome analysis and were analyzed using the Seurat R package (version 4.1.0). The data were deposited in PubMed under GSE239612.

### Flow cytometric analysis of human PBMCs and hepatic mononuclear cells

Cryopreserved human PBMCs and hepatic mononuclear cells were thawed and then incubated with fluorochrome-conjugated monoclonal antibodies for 30 min at 4 °C. The cells were then preincubated with anti-CD16/32 Fc blocker (BD Pharmingen, Franklin Lakes, NJ, USA), which was followed by staining with the Live/Dead marker anti-FVD-APC-Cy7 (all supplied by eBioscience, San Diego, CA, USA). The fluorochrome-conjugated antibodies used were anti-CD3-PerCP-Cy5.5, anti-CD3-PE-Cy7, anti-CD4-AF700, anti-CD8-PE, anti-CD8-APC, anti-CD28-APC, anti-CD57-FITC, anti-IFN-γ-PE-Cy7, anti-TNF-α-APC, anti-IL-17A-APC, anti-perforin-PerCP-Cy5.5, and anti-granzyme B-PE (all supplied by eBioscience). Following this, the PBMCs and hepatic mononuclear cells were stimulated with phorbol-myristate acetate/ionomycin/brefeldin A/monensin for 5 h ex vivo, after which they were fixed and permeabilized using a Fixation/Permeabilization Buffer kit (eBioscience). The permeabilized cells were washed with FACS buffer; resuspended in 1% formaldehyde; and immunostained for intracellular cytokines using anti-IFN-γ-PE-Cy7, anti-TNF-α-APC, anti-IL-17A-APC, anti-perforin-PerCP-Cy5.5, and anti-granzyme B-PE fluorochrome-conjugated antibodies. The stained cells were analyzed using a BD LSRFortessa flow cytometer (BD Biosciences, San Jose, CA, USA), and the data were analyzed using FlowJo software (Treestar, Ashland, OR, USA).

### Single-cell transcriptomic analysis

A gene-cell matrix for single-cell transcriptomics was downloaded from the GEO (GSE159977 and E-MTAB-10553) [15, 16], and the output of the count matrix was read using the Read10X function in the Seurat package (Version 4.1.0) and the read.table function; the latter was further converted to dgCMatrix format. The outputs of the count matrix were read using the Read10X function from the Seurat package (Version 4.1.0) and the read.table function, and the latter was further converted to dgCMatrix format. After generating the feature-barcode matrix, we discarded cells with total unique molecular identifier count <500. To exclude low-quality cells from the data, we filtered out those in which mitochondrial genes accounted for >20% of the total number. We excluded outlier cells from the downstream analysis using the isOutlier function in the scater R package. A global-scaling normalization method (“LogNormalize”) was employed to ensure that the total gene transcript expression in each of the cells was similar, and the scaling factor was set to 10,000. The top 2000 differentially expressed genes were subjected to downstream analysis using the FindVariableFeatures function. The ScaleData function, “vars.to.regress” option UMI, and percentage mitochondrial gene content were used to remove unwanted sources of variation.

Principal components analysis (PCA), incorporating highly variable features, was used to reduce the dimensionality of this dataset, and the first 30 PCs were identified for analysis. FindClusters using shared neighbor module optimization was then used, based on the first 17 PCs with a clustering resolution of 0.1, to create eight initial clusters. Most of the parameters we tried produced similar UMAP clustering, but the use of 17 PCs was associated with the best separation between different cell types. For each cell type, a marker gene was identified using the Seurat function FindAllMarkers and MAST. All analyses were performed in the Seurat R package (version 4.1.0) [16].

### Single-nucleus RNA-sequencing data analysis

Single-nucleus RNA-sequencing (snRNA-seq) data analysis was performed on the mouse dataset GSE212837 [17]. The Cell Ranger’s filtered feature-barcode metrics were initially used for quality control (QC) and subsequently processed through the standard Seurat pipeline in the research paper where the data was utilized. Distinct QC parameters were applied for snRNA-seq data obtained using the Chromium 3’ Gene Expression V2 kit with Seurat for the mouse sample. Nuclei satisfying the criteria of total counts greater than 500 and less than 15,000 were included in the analysis. Upon downloading the mtx and two tsv files resulting from the Cell Ranger analysis, LIGER was utilized for the analysis, in contrast to scRNA-seq data analysis, as mentioned in the paper. A LIGER object was created by grouping cells based on the Ctrl and NASH conditions using the createLiger function. The optimizeALS function was then employed with K = 10 and resolution = 0.2. Differential gene expression analysis was conducted using the Wilcoxon rank sum test to identify cluster-specific gene expression in each nucleus and against all other nuclei. Cell types were manually assigned based on differentially expressed genes. The reduced dimensional data was visualized using the principal components previously used for clustering and UMAP representation.

### Statistical analysis

Continuous datasets are reported as the mean ± SEM, except if explicitly stated otherwise. Statistical analyses were performed using Prism software version 8.3.0 (GraphPad, San Diego, CA, USA), after confirming the normal distribution of each dataset. The ‘ggstatsplot’ creates graphics with details from statistical tests included in the plots themselves. Spearman’s correlation was used to analyze the relationships between normally distributed variables. Statistical analyses were performed using R software version 4.1.0 (R project for Statistical Computing, Vienna, Austria), and *P* < 0.05 was considered to indicate statistical significance.

## Results

### Characteristics of the participants

Fifty-nine participants with type 2 diabetes, aged 36–75 years (58.7 ± 11.0 years) and with a BMI between 19.6 and 32.6 kg/m^2^ (26.1 ± 2.9 kg/m^2^), were enrolled in the study between July 2019 and March 2020 (Supplementary Table 1). Among the participants, 37 had obesity (BMI ≥ 25 kg/m^2^), 15 had overweight (BMI 23–25 kg/m^2^), and 7 had normal weight (BMI 18–23 kg/m^2^). Twenty-eight (47.5%) of the participants were male. Their mean fasting plasma insulin, C-peptide, and glucose concentrations were 10.7 ± 9.7 μIU/L, 2.1 ± 1.1 ng/dL, and 146.1 ± 44.6 mg/dL, respectively. The mean HOMA-IR of the participants was 3.96 ± 4.28, and 31 (52.5%) participants had values > 2.5 (Supplementary Table 1).

Liver MRI and ultrasonography were used to quantitatively assess the hepatic steatosis and fibrosis of the participants with T2D. The MRI-PDFF and mean liver stiffness values obtained using MR elastography were 1.23–35% (10.2% ± 7.2%) and 1.3–3.8 kPa (2.0 ± 0.4 kPa), respectively (Supplementary Table 1). The median and mean ATI values, obtained using 2D-shear-wave elastography, were 0.46–1.02 dB/cm/MHz (0.72 ± 0.12 dB/cm/MHz) and 4.5–12.2 (6.86 ± 1.60 kPa), respectively (Supplementary Table 1). The aspartate aminotransferase-to-platelet ratio, nonalcoholic fatty liver disease liver fat score, hepatic steatosis index, NAFLD fibrosis score, and fibrosis-4 index were 0.12–0.88 (0.28 ± 0.14), −3.60–8.21 (0.79 ± 0.14), 26.9–48.5 (37.6 ± 4.74), −1.35–5.54 (1.73 ± 1.03), and 0.42–3.04 (1.32 ± 0.55), respectively (Supplementary Table 1).

### T-cell senescence is associated with insulin resistance and liver fibrosis in patients with type 2 diabetes

**Fig. 1:**
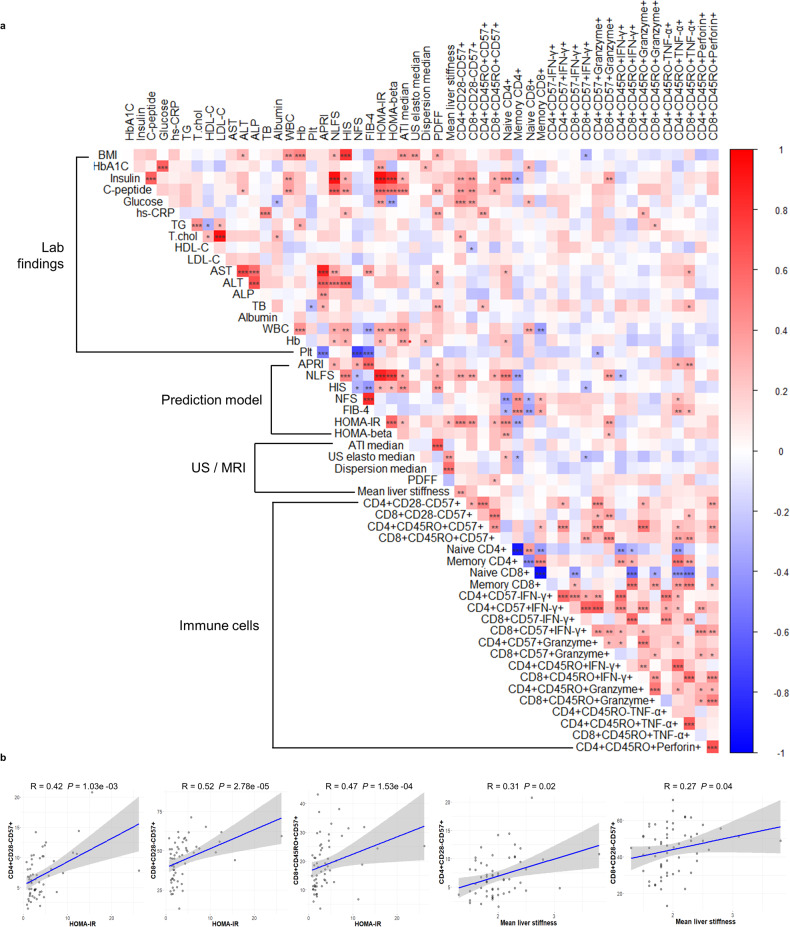
Correlation analysis of variables identified as significant in linear regression analyses of data for participants with type 2 diabetes. **a** Correlogram, with the depth of shading representing the magnitude of the correlation and positive and negative correlations being shown in blue and red, respectively. **b** Relationships of circulating senescent T cells with insulin resistance or liver stiffness in participants with T2D, evaluated using Spearman’s correlation analysis. BMI body mass index, HbA1c hemoglobin A1c, hs-CRP high-sensitivity C-reactive protein, TG triglyceride, T Chol total cholesterol, HDL-C high-density lipoprotein-cholesterol, LDL-C low-density lipoprotein-cholesterol, AST aspartate aminotransferase, ALT alanine aminotransferase, ALP alkaline phosphatase, TB total bilirubin, WBC white blood cell, PLT platelet, APRI aspartate aminotransferase-to-platelet ratio, NLFS nonalcoholic fatty liver disease liver fat score, HIS hepatic steatosis index, NFS nonalcoholic fatty liver disease fibrosis score, FIB-4 fibrosis-4 index, HOMA-IR homeostatic model assessment-insulin resistance, HOMA-beta homeostasis model assessment-β-cell function, ATI attenuation imaging, US ultrasonography, PDFF proton density fat fraction, CD cluster of differentiation, IFN-γ interferon gamma, TNF tumor necrosis factor.

To evaluate the relationships of systemic inflammation with metabolic profile and hepatic dysfunction, we characterized the immunophenotype of the T cells in the PBMCs obtained from participants with T2D using FACS analysis. Participants with high HOMA-IR not only had higher fasting insulin, C-peptide, and glucose concentrations but also had higher NLFS and HIS scores (Supplementary Table 2). We also found that the participants with high HOMA-IR had significantly higher levels of liver fibrosis, assessed using MR elastography (Fig. 1a, Supplementary Fig. 1, and Supplementary Table 2). Moreover, there was a significant relationship between the quantity of liver fat, assessed using MRI-PDFF, and the number of CD8 + CD45RO + CD57 + T cells in participants with T2D (Fig. 1a and Table 1). In addition, the HOMA-IR and mean liver stiffness were positively associated with T-cell senescence in participants with T2D (Fig. 1b).

**Table 1:** Results of the correlation analysis of the relationships of metabolic parameters with T-cell subtypes and senescent T-cell populations in participants with nonalcoholic type 2 diabetes (*n* = 59).

	Naïve CD4^+^	Memory CD4^+^	Naïve CD8^+^	Memory CD8^+^	CD4^+^CD28^−^CD57^+^	CD8^+^CD28^−^CD57^+^	CD4^+^CD45RO^+^ CD57^+^	CD8^+^CD45RO^+^ CD57^+^	CD4 + CD45RO+ TNF-α +	CD8 + CD45RO+ TNF-α +	ATI median value	PDFF	Mean liver stiffness
Age	−0.365* (0.005)	0.333* (0.010)	−0.593* (0.000)	0.600* (0.000)	−0.159 (0.228)	0.166 (0.209)	0.068 (0.609)	0.119 (0.369)	0.112 (0.397)	0.241 (0.066)	−0.200 (0.129)	−0.219 (0.096)	−0.168 (0.203)
Body mass index	0.252* (0.054)	−0.174 (0.188)	0.007 (0.957)	0.015 (0.913)	0.118 (0.374)	−0.006 (0.964)	0.070 (0.600)	0.143 (0.281)	−0.104 (0.431)	0.159 (0.228)	0.395* (0.002)	0.332* (0.010)	0.166 (0.210)
HbA1c	0.086 (0.517)	−0.119 (0.371)	0.288* (0.027)	−0.250 0.057	0.245 (0.062)	0.216 (0.101)	−0.069 (0.603)	−0.067 (0.616)	−0.211 (0.109)	−0.103 (0.437)	−0.007 (0.957)	0.111 (0.404)	0.089 (0.503)
Fasting insulin	0.422* (0.001)	−0.315* (0.015)	0.139 (0.295)	−0.113 (0.396)	0.336* (0.009)	0.389* (0.002)	−0.016 (0.905)	0.315* (0.015)	−0.075 (0.572)	0.140 (0.291)	0.287* (0.027)	0.160 (0.225)	0.231 (0.078)
Fasting c-peptide	0.124 (0.350)	−0.110 (0.408)	0.037 (0.784)	−0.013 (0.921)	0.372* (0.004)	0.395* (0.002)	0.188 (0.154)	0.324* (0.012)	−0.101 (0.447)	0.107 (0.418)	0.468* (0.000)	0.344* (0.008)	0.110 (0.408)
Fasting glucose	0.160 (0.227)	−0.179 (0.176)	0.276* (0.034)	−0.205 (0.119)	0.424* (0.001)	0.372* (0.004)	0.117 (0.379)	0.094 (0.480)	−0.050 (0.708)	−0.028 (0.832)	−0.009 (0.947)	−0.056 (0.674)	0.236 (0.072)
hs-CRP	−0.032 (0.812)	0.048 (0.716)	−0.077 (0.562)	0.092 (0.487)	0.180 (0.173)	0.122 (0.358)	0.357* (0.006)	−0.001 (0.992)	0.033 (0.802)	0.143 (0.281)	−0.006 (0.964)	0.358* (0.005**)**	0.018 (0.892)
HOMA-IR	0.423* (0.001)	−0.339* (0.009)	0.197 (0.135)	−0.169 (0.201)	0.457* (0.000)	0.402* (0.002)	−0.046 (0.732)	0.292* (0.025)	−0.112 (0.398)	0.063 (0.636)	0.262* (0.045)	0.124 (0.348)	0.280* (0.032)
HOMA- β	0.337* (0.009)	−0.206 (0.117)	0.036 (0.786)	−0.013 (0.924)	0.110 (0.406)	0.227 (0.084)	0.026 (0.846)	0.243 (0.063)	−0.027 (0.841)	0.198 (0.134)	0.249 (0.057)	0.180 (0.172)	0.143 (0.280)
Triglycerides	0.079 (0.551)	−0.043 (0.744)	0.054 (0.683)	−0.046 (0.731)	0.093 (0.483)	0.112 (0.399)	−0.031 (0.817)	0.175 (0.185)	0.007 (0.958)	0.005 (0.967)	0.178 (0.177)	0.210 (0.110)	−0.109 (0.412)
Total cholesterol	0.079 (0.387)	−0.017 (0.896)	0.002 (0.991)	−0.025 (0.849)	0.324* (0.012)	−0.010 (0.938)	0.106 (0.424)	0.143 (0.280)	0.137 (0.302)	0.005 (0.748)	−0.027 (0.839)	0.065 (0.626)	0.024 (0.855)
NLFS	0.493* (0.000)	−0.358* (0.005)	0.057 (0.667)	−0.037 (0.779)	0.343* (0.008)	0.349* (0.007)	0.002 (0.985)	0.322* (0.013)	−0.062 (0.640)	0.226 (0.085)	0.324* (0.012)	0.291* (0.026)	0.190 (0.149)
HIS	0.242 (0.064)	−0.221 (0.092)	0.031 (0.816)	0.017 (0.897)	0.198 (0.132)	−0.014 (0.915)	0.130 (0.325)	0.118 (0.373)	−0.062 (0.643)	0.154 (0.245)	0.410* (0.001)	0.403* (0.002)	0.014 (0.917)
NFS	−0.389* (0.002)	0.415* (0.001)	−0.284* (0.030)	0.263* (0.044)	−0.024 (0.856)	0.025 (0.849)	0.056 (0.675)	0.100 (0.453)	0.262* (0.045)	0.174 (0.188)	−0.174 (0.188)	−0.175 (0.185)	0.141 (0.288)
FIB-4	−0.294* (0.024)	0.423* (0.001)	−0.361* (0.005)	0.323* (0.013)	−0.069 (0.604)	−0.009 (0.945)	0.035 (0.792)	0.170 (0.198)	0.354* (0.006)	0.287* (0.027)	−0.182 (0.168)	−0.065 (0.626)	0.046 (0.728)

*hs-CRP* high-sensitivity C-reactive protein, *HOMA-IR* homeostatic model assessment-insulin resistance, *HOMA-β* homeostatic model assessment-β-cell function, *NLFS* nonalcoholic fatty liver disease liver fat score, *HIS* hepatic steatosis, *NFS* nonalcoholic fatty liver disease fibrosis score, *FIB-4* fibrosis-4 index.

**Fig. 2:**
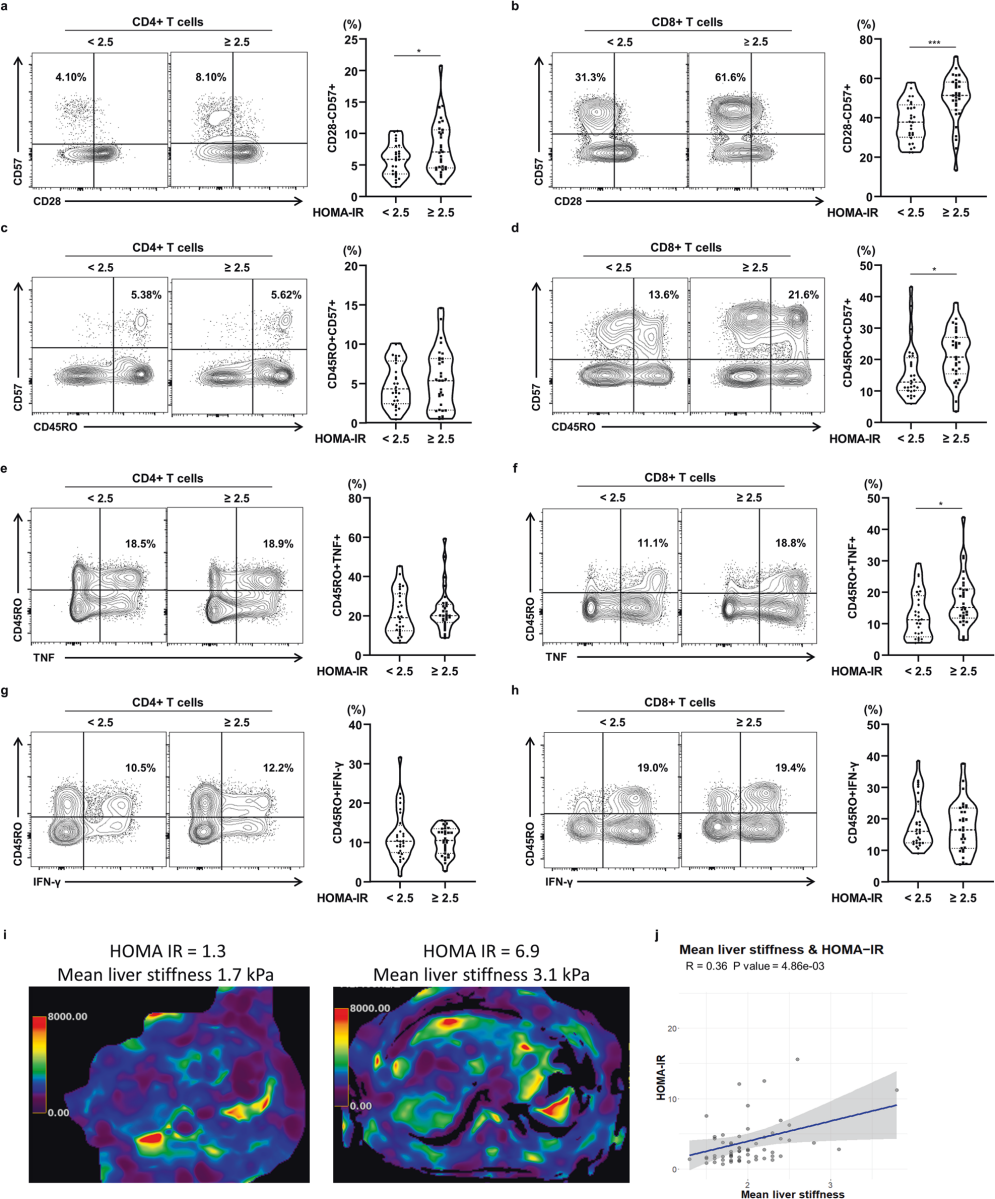
Immunophenotypic characteristics of peripheral blood mononuclear cells in participants with T2D. **a**, **b** Percentages of CD28–CD57+ cells and **c**, **d** percentages of CD45RO + CD57+ cells within the CD4+ and CD8 + T-cell population of T2D patients. **e**, **f** Percentage of TNF-producing CD45RO+ cells within the CD4+ and CD8 + T-cell population. **g**, **h** percentage of IFN-γ-producing CD45RO+ cells within the CD4+ and CD8 + T-cell population. **i** Color MRE, showing the liver stiffness of participants with T2D and low or higher HOMA-IR. **j** Association between mean liver stiffness and HOMA-IR in participants with T2D (*n* = 59). Asterisks indicate significant differences between participants with T2D and HOMA-IR < 2.5 or ≥2.5. The unpaired *t*-test was used to analyze the data. *P < 0.05, ***P < 0.001. Data are presented as the mean ± SEM. HOMA-IR homeostasis model assessment-insulin resistance, IFN-γ interferon gamma, TNF tumor necrosis factor, MRE magnetic resonance elastography.

To quantify the T-cell senescence in peripheral blood samples from participants with T2D, we counted the numbers of CD57+ and/or CD28 − T cells in the CD4+ and CD8 + T-cell populations of the obtained PBMCs. Based on the previous studies, human senescent CD8 + T cells are associated with development of T2D [10, 18]. In the current study, we also found an increase in the senescent CD8 + T cells in the patients with type 2 diabetes compared to the control subjects without T2D (Supplementary Fig. 2a, b). Participants with high HOMA-IR had significantly larger populations of CD28 − CD57+ senescent T cells among the CD4+ and CD8 + T cells than those with lower HOMA-IR (Fig. 2a, b and Supplementary Table 2). Although the size of the population of CD4 + CD45RO + CD57+ cells showed no relationship with insulin resistance status in the participants with T2D (Fig. 2c), the number of CD8 + CD45RO + CD57+ cells was much higher in patients with high HOMA-IR (Fig. 2d and Supplementary Table 2). However, we found that there was no significant relationship between HOMA-beta and T-cell senescence in patients with T2D. We also investigated the association of additional metabolic markers including lipid profiles and adipokines with T-cell senescence in patients with T2D. HDL levels were negatively correlated with senescent CD8 + T cells, but Total cholesterol levels were positively associated with senescent CD4 + T cells in this cohort (Fig. 1a). Moreover, we showed the low serum adiponectin levels in the T2D patients with high HOMA-IR, and T2D patients with higher population of senescent CD8 + T cells exhibited low serum adiponectin levels (Supplementary Fig. 3a, b). However, HOMA-beta was not significantly associated with senescent T cells in T2D patients (Supplementary Fig. 3c). Moreover, there was no significant correlation between renal function markers and T-cell senescence in patients with type 2 diabetes (Supplementary Fig. 3c).

Senescent T cells produce larger quantities of proinflammatory cytokines, leading to tissue inflammation and insulin resistance [19]. Therefore, we investigated the functional characteristics of the circulating CD4+ and CD8 + T cells obtained from participants with T2D. TNF-α expression was significantly higher in memory CD8 + T cells, but not in memory CD4 + T cells, from participants with high HOMA-IR (Fig. 2e, f). However, the expression of IFN-γ in senescent CD4+ and CD8 + T cells was not related to the insulin resistance status of the participants with T2D (Fig. 2g, h). The HOMA-IR was also found to be positively associated with liver stiffness (Fig. 2i, j). We have also incorporated a multivariate analysis in our study, specifically adjusting for age, BMI, and sex, to investigate the association of HOMA-IR with various confounding factors of T-cell senescence (Supplementary Fig. 4a–l). Taking these findings together, insulin resistance is associated with both T-cell senescence and TNF-α production by senescent CD8 + T cells, and correlates with the degree of hepatic fibrosis in patients with T2D.

To further investigate the relationship of obesity with the immunophenotype of T cells in participants with T2D, we analyzed the CD28 − CD57+ senescent subset of the CD4+ and CD8 + T cells using flow cytometry. The numbers of senescent CD4+ and CD8 + T cells and CD45RO + CD57+ cells in the CD4+ and CD8 + T-cell populations were not associated with the presence of obesity in participants with T2D (Supplementary Fig. 5a–d). In addition, the numbers of TNF-α- or IFN-γ-producing memory CD4+ and CD8 + T cells did not significantly differ in participants with T2D and lower or higher BMI (Supplementary Fig. 5e–h). These data suggest that obesity is not closely associated with T-cell senescence or the inflammatory response in patients with T2D.

### T-cell senescence and exhaustion in the liver may be involved in NASH and liver cirrhosis

**Fig. 3:**
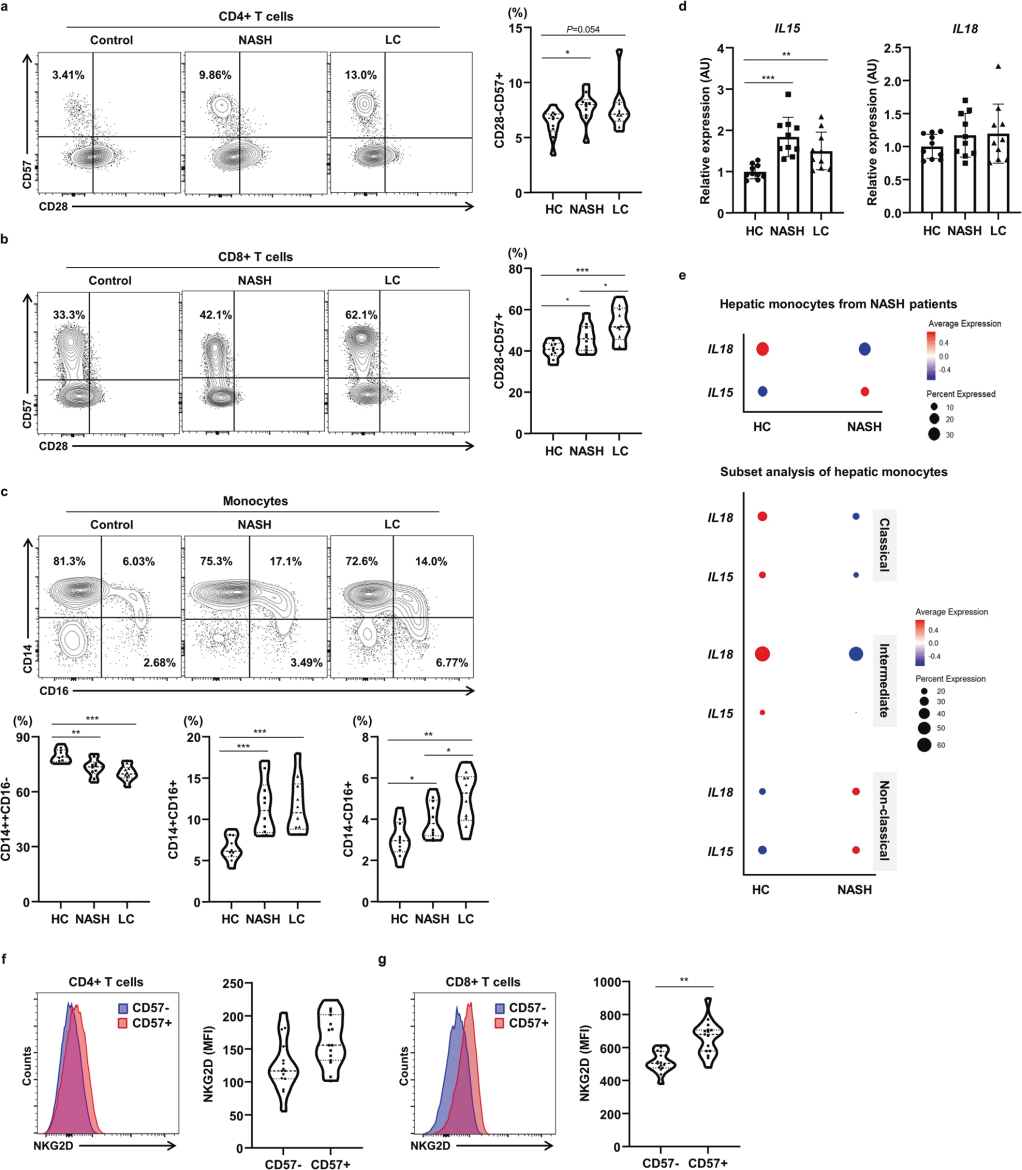
Hepatic T-cell senescence and exhaustion in participants with T2D and with or without NASH or liver cirrhosis. **a**, **b** Percentages of CD28–CD57+ cells within the CD4+ and CD8 + T-cell population, and **c** percentages of hepatic classical (CD14 + + CD16 − ), intermediate (CD14 + + CD16 + ), and nonclassical (CD14 + CD16 + +) monocytes in participants with T2D and with or without NASH or liver cirrhosis. **d** *IL-15* and *IL18* expression in hepatic monocytes from participants with T2D and with or without NASH or liver cirrhosis. **e** Gene expression of *IL-15* and *IL18* in hepatic monocytes and their subsets, analyzed using hepatic single-cell transcriptomic data from the Gene Expression Omnibus (GSE159977 and E-MTAB-10553). The size of each dot represents the percentage expression of a specific gene, compared to that of all other transcripts, and the color gradient of the dot indicates the mean expression of the gene. **f** Expression of NKG2D, quantified using the median fluorescence intensity in hepatic CD57+ or CD57− cells within the CD4+ and CD8 + T-cell population of participants with T2D and with or without NASH or liver cirrhosis. Data are presented as the mean ± SEM. Data in **a**–**d** were analyzed using one-way ANOVA, and those in **f** and **g** were analyzed using the unpaired *t*-test. *P < 0.05, **P < 0.01, ***P < 0.001. HC healthy controls, NASH nonalcoholic steatohepatitis, LC liver cirrhosis, IL interleukin, NKG2D natural killer group 2D, MFI median fluorescence intensity.

Because of the relationship between insulin resistance and liver fibrosis identified in the participants with T2D, we further characterized the immunophenotypes of liver-infiltrating T cells in participants with T2D and NASH or liver cirrhosis using flow cytometry (Supplementary Table 3). Control subjects were regarded as T2D participants without advanced liver diseases such as NASH, liver cirrhosis, or liver cancer. As shown in Fig. 3a, b, the participants with T2D and NASH or liver cirrhosis had greater numbers of CD28 − CD57+ senescent CD4+ and CD8 + T cells in their livers than the healthy controls. Interestingly, the numbers of CD14 + + CD16+ intermediate and CD14 + CD16 + + nonclassical monocytes were greater in the livers of participants with T2D and NASH or liver cirrhosis than in those of participants with T2D but no NASH or cirrhosis (Fig. 3c).

Because of the possibility of interleukin (IL) 15-mediated bystander activation of T-cell senescence, we measured IL-15 expression in liver mononuclear cells from participants with NASH and liver cirrhosis. We found that *IL-15* expression was significantly higher in the hepatic mononuclear cells from participants with NASH or liver cirrhosis than in those from controls, but *IL18* expression did not significantly differ among the three groups (Fig. 3d). We also analyzed the single-cell RNA-sequencing data for monocytes obtained from healthy controls and patients with NASH [16], and found higher *IL-15* expression in monocytes from participants with NASH. In addition, the nonclassical monocytes from participants with NASH showed higher expression of both *IL-15* and *IL18* (Fig. 3e). Moreover, *IL2B* and *IL15RA* expression in hepatic monocytes was higher in participants with NASH than in healthy controls (Supplementary Fig. 6a). Consistent with this high monocytic IL-15 expression causing bystander activation of CD8 + T cells [20], NKG2D expression by hepatic CD8 + T cells was significantly higher alongside T-cell senescence in participants with T2D and NASH (Fig. 3f, g). Moreover, the numbers of PD-1 + CD4+ and CD8 + T cells increased with the progression of liver disease in the participants with T2D (Supplementary Fig. 6b). Collectively, these data suggest that hepatic T cells with senescence and IL-15-producing nonclassical monocytes may be involved in the progression of liver disease in patients with T2D.

### Senescent and exhausted T cells from the livers of patients with NASH or liver cirrhosis show abnormal expression profiles

**Fig. 4:**
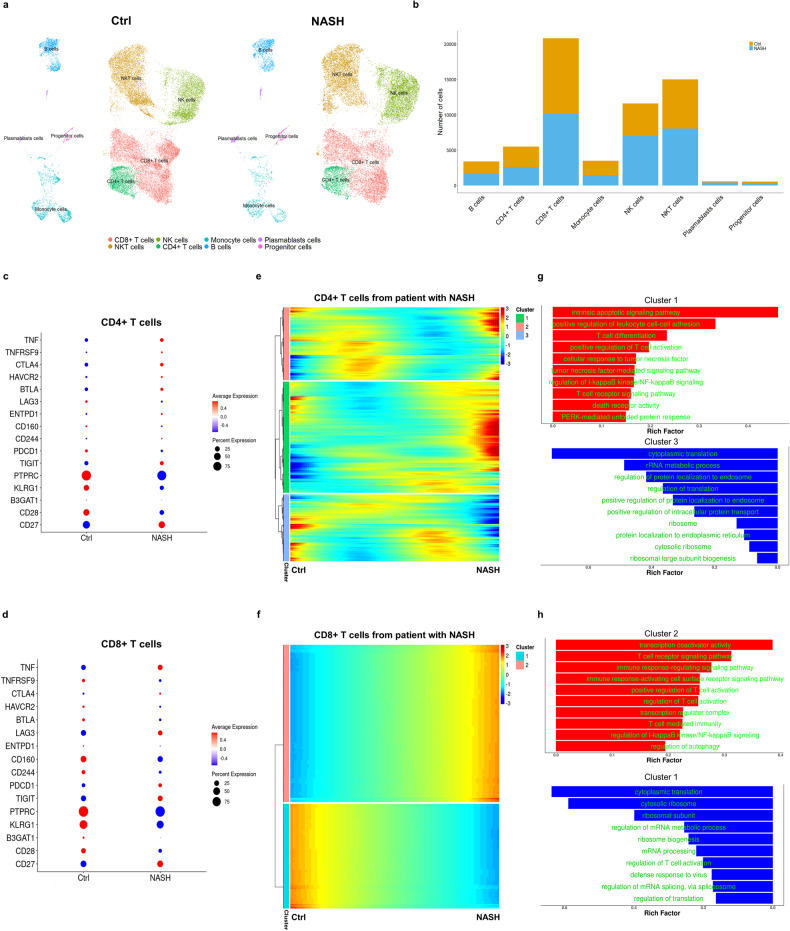
Hepatic single-cell transcriptomic data analysis of patients with NASH. Liver samples were analyzed to better characterize the tissue-infiltrating immune cells. Approximately 60,925 single cells from participants with NASH (*n* = 3) and healthy controls (*n* = 4) were subjected to single-cell transcriptomic analysis. **a** Single-cell transcriptome data-based UMAP representation showing the FlowSOM-guided clustering of CD45+ cells in participants with or without NASH. Colors are used to differentiate annotated cell types. **b** Bar plot showing the relative contributions of hepatic cells from participants with or without NASH. **c**, **d** Dot plots showing the marker genes of senescence and exhaustion identified in CD4+ and CD8 + T cells. The size of the dot represents the proportion of the cell population that expresses each gene, and the color indicates the level of expression. **e**, **f** Gene expression of hepatic CD4+ and CD8 + T cells from participants with NASH and healthy controls, analyzed along a latent time/pseudotime axis corresponding to the pathological progression of each cell. **g**, **h** Bar plots showing pathway enrichment analysis. Rich factor: ratio of the expression of a differentially expressed gene annotated in this pathway term to that of all the genes annotated in this pathway term. A higher rich factor implies higher expression.

Next, to characterize the relationship of T-cell phenotype with the advanced stages of liver disease, including NASH [16] and liver cirrhosis [11], we separately analyzed the distinct cell types (Fig. 4a, b and Supplementary Fig. 7) natural killer (NK) cells, T cells, monocyte-derived macrophages, Kupffer cells, hepatocytes, cholangiocytes, and hepatic stellate cells (HSCs) from the livers of healthy controls and participants with NASH using single-cell transcriptomic data (Fig. 4a). There were no differences in the numbers of liver immune cells in healthy individuals and participants with NASH (Fig. 4b). Therefore, to better understand the immune microenvironment of the livers, we profiled the gene expression of hepatic CD4+ and CD8 + T cells from controls and participants with NASH. We found significantly higher expression of genes related to senescence (e.g., PTPRC, TIGIT, and TNF) and exhaustion (e.g., PDCD1, CTLA4, LAG3, and TNFRSF9) in CD4+ and CD8 + T cells from participants with NASH (Fig. 4c, d), implying an association between T-cell dysfunction and the progression of liver disease in humans.

We next performed pseudotime trajectory analysis on hepatic CD4+ and CD8 + T cells from participants with NASH and healthy controls. The major T-cell populations were defined using the expression of marker genes in hepatic CD4+ and CD8 + T cells (Fig. 4e, f), and we evaluated the regulation of gene expression during disease progression and the respective functions of the up- and downregulated genes related to T-cell senescence and exhaustion. We found significantly higher expression of markers of T-cell senescence (e.g., PTPRC, TIGIT, and TNF) and exhaustion (e.g., PDCD1, CTLA4, LAG3, and TNFRSF9) in hepatic CD4+ and CD8 + T cells in participants with NASH or liver cirrhosis than in controls (Supplementary Fig. 8a, b). Next, we sought to identify the relevant biological functions of the up- or downregulated genes in the form of GO biological pathways. Cluster 1 and cluster 3 in the hepatic CD4 + T cells were highly enriched in the NASH and control groups, respectively (Fig. 4e). Apoptotic signaling pathways, T-cell activation, and TNF-mediated signaling were more closely associated with cluster 1 (Fig. 4g) and may have contributed to the progression of hepatic inflammation. Cluster 2 in the hepatic CD8 + T cells was highly enriched in participants with NASH, and this was associated with transcription coactivator activity, T-cell receptor signaling, and T-cell-mediated immunity (Fig. 4h).

Participants with liver cirrhosis had fewer Kupffer cells, NK cells, quiescent HSCs, and T cells, but more activated HSCs, mesothelial cells, and vascular endothelial cells, in their livers (Supplementary Fig. 9a, b). This indicates that there are alterations in the populations of hepatic nonparenchymal cells during the scarring of the liver that is induced by long-term damage. Genes related to senescence (e.g., PTPRC, TIGIT, and TNF) and exhaustion (e.g., PDCD1, CTLA4, LAG3, and TNFRSF9) were highly enriched in CD4+ and CD8 + T cells from the participants with liver cirrhosis (Supplementary Fig. 9c, d). Three major T-cell populations were defined on the basis of the expression of marker genes in hepatic CD4+ and CD8 + T cells (Supplementary Fig. 9e–h), which demonstrates the dynamics of hepatic T-cell fate determination related to senescence and exhaustion in the livers of patients with liver cirrhosis (Supplementary Fig. 10a, b).

### Hepatic T-cell senescence and exhaustion are implicated in the mouse model of NASH

**Fig. 5:**
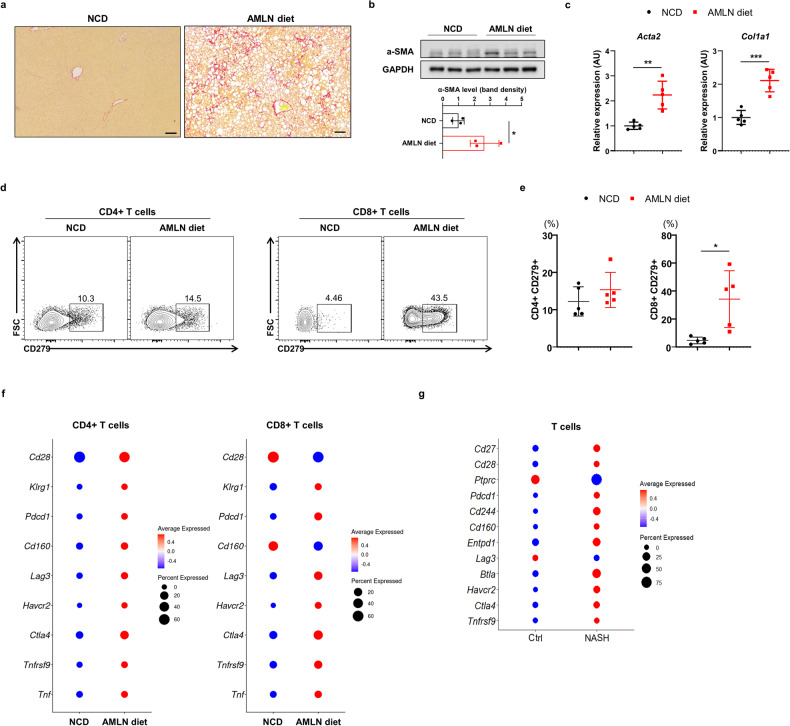
Mouse model with NASH exhibits higher induction of the markers of senescence and exhaustion in the liver-infiltrating T cells. **a** Sirius red staining of liver sections from the mice fed a NCD or AMLN diet for 30 weeks. **b** Western blot analysis for α-SMA in the liver tissues and graphs showing quantification of band density of α-SMA protein in the liver tissues (normalized to GAPDH) in the mice fed a NCD or AMLN diet for 30 weeks. **c** Real-time RT-PCR analysis of *Acta2* and *Col1a1* in whole liver cDNA. **d**, **e** FACS analysis of hepatic CD4 + CD279+ and CD8 + CD279 + T cells isolated from mice fed a NCD or AMLN diet for 30 weeks. **f** Dot plots showing the marker genes of senescence and exhaustion identified in CD4+ and CD8 + T cells from liver mononuclear cells from mice fed a NCD or AMLN diet for 30 weeks. The size of the dot represents the proportion of the cell population that expresses each gene, and the color indicates the level of expression. The data were deposited in the Gene Expression Omnibus (GEO) database under accession number GSE239612. **g** Dot plots showing the marker genes of senescence and exhaustion identified in T cells from control and chemical-induced murine NASH model. The data were deposited in the Gene Expression Omnibus (GEO) database under accession number GSE212327. Data are presented as the mean ± SEM. Data in **b**, **c**, **e** were analyzed using the unpaired *t*-test. *P < 0.05, **P < 0.01, ***P < 0.001. NCD normal chow diet, AMLN Amylin liver NASH, Ctrl control.

Next, to clarify the association of T-cell senescence and exhaustion with NASH development, we successfully generated animal models of NASH by employing an Amylin Liver NASH (AMLN) diet consisting of 40% high-fat content, 22% high-fructose content, approximately 18% trans-fatty acids, and 2% high-cholesterol for 30 weeks. As shown in Fig. 5a, collagen deposition was remarkably increased in the livers of mice fed AMLN diet. Moreover, we demonstrated an increase in α–SMA expression in the livers of NASH mouse model, indicating hepatic fibrosis (Fig. 5b). Next, to conduct a comprehensive analysis of T-cell senescence and exhaustion in an animal model with NASH, we utilized flow cytometry and hepatic single-cell transcriptomics in the C57BL/6 J mice fed with NCD or AMLN diets for 30 weeks. We separately analyzed the hepatic distinct cell types using unsupervised UMAP analysis (Supplementary Fig. 11). Similar to previous results of higher enrichment of hepatic T-cell senescence and exhaustion in mice with diet-induced obesity, we observed the induction of exhaustion in liver-infiltrating T cells within the NASH mouse model using FACS analysis (Fig. 5c, d). Additionally, in hepatic single-cell transcriptomic analysis, the markers of senescence and exhaustion were highly expressed in the liver-infiltrating CD4+ and CD8 + T cells of mice with NASH (Fig. 5e). This association of T-cell senescence and exhaustion with NASH was also validated in another single-cell transcriptome dataset [17] from chemical-induced mouse model with NASH (Fig. 5f). These findings suggest that hepatic T-cell senescence and exhaustion are also implicated in the development of NASH in murine model.

## Discussion

Insulin resistance, a hallmark of T2D, is closely associated with inflammation in insulin-target tissues such as the liver, adipose tissue, and skeletal muscle [21]. The T2D-associated low-grade systemic inflammatory response plays an important role in the development of NASH and liver fibrosis [22]. Here, we have shown that patients with insulin resistance or T2D are not only more likely to develop fatty liver disease, including NASH and liver fibrosis, but also demonstrate systemic immunosenescence and inflammation. We also found that patients with NASH or liver cirrhosis have more marked hepatic T-cell senescence and exhaustion, which may promote the progression of metabolic liver disease in humans.

A balance is required between effector and tolerogenic immune responses to ensure hepatic and whole-body homeostasis [23]. It is known that T-cell senescence and exhaustion play a key role in the deterioration of tissue homeostasis and lead to immunopathological outcomes during viral infection and autoimmune disease [19]. In the present study, we have shown the clinical relevance of T-cell senescence and exhaustion for the spectrum of metabolic liver disease and insulin resistance in humans, using PBMC immunophenotyping using flow cytometry and single-cell transcriptomic analysis of the liver. Although T cells become less able to respond to viral infection and cancer with time, as an inevitable consequence of aging, the relationship between T-cell senescence and metabolic liver disease remains to be fully characterized. We have shown that insulin resistance-associated CD8 + T-cell senescence is more marked in patients with T2D and liver fibrosis (Figs. 1 and 2). In addition, hepatic T cells were shown to exhibit high expression of markers of senescence and exhaustion in patients with NASH or liver cirrhosis (Fig. 3). Conversely, NASH has been shown to impair metabolic fitness and the motility of hepatic CD8 + T cells in a mouse model of NASH-induced liver cancer, and these are restored by metformin treatment [24]. These results imply a role for T-cell immunity in the induction of insulin resistance-associated hepatic inflammatory disease.

T-cell senescence increases during aging and in inflammatory diseases, which is at least in part because of lifelong antigen activation by persistent viruses such as cytomegalovirus and Epstein-Barr virus [25]. Although senescent T cells have low proliferative capacity and are considered to be dysfunctional [26], they have potent cytotoxic activity and secrete proinflammatory cytokines such as TNF-α and IFN-γ following their activation. This senescence-associated secretory phenotype induces the progression of tissue inflammation [10]. In the present study, we found that *IL-15* expression is high in the hepatic monocytes of patients with NASH or liver cirrhosis. IL-15-mediated bystander activation has been implicated in aging and inflammatory diseases, an effect that is mediated through the expression of NK receptors on senescent T cells [20], and contributes to the immunopathology through antigen-independent activation. IL-15 is also implicated in the expansion of CD8 + T cells with a memory phenotype [27]. It has been shown to be required for diet-induced insulin resistance and NASH in mice [28, 29], and its expression also positively correlates with HOMA-IR in patients with autoimmune diabetes [30]. Moreover, IL-15 is involved in the progression of liver fibrosis by activating HSCs in patients with viral hepatitis [31]. These findings suggest that the metabolic disease-associated hepatic inflammation signature may be the result of bystander cytokine-mediated T-cell senescence and exhaustion. However, it is also important to better understand how bystander cytokine-activated senescent T cells interact with hepatic parenchymal or nonparenchymal cells and affect hepatic immunopathology as part of the spectrum of metabolic liver disease.

HSCs play a key role in the development and progression of fibrosis by producing large amounts of extracellular matrix components such as type 1 collagen [32]. HSCs are activated by proinflammatory cytokines and chemokines in chronic liver injury and interact with other liver-resident cells, thereby affecting hepatic function and immunopathology [33]. Thus, it is not surprising that key proinflammatory cytokines, including TNF-a, IL-6, CCL2, and CCL5, which are present at high serum concentrations in patients with insulin resistance, are mediators of HSC activation [32, 33]. Data from the National Health and Nutrition Examination Survey indicate that insulin resistance is associated with a high risk of liver fibrosis in patients with hepatic steatosis [34]. Moreover, hyperinsulinemia has also been shown to be a key factor in the progression of fibrosis in patients with NASH [35]. Although we have shown relationships of T-cell senescence and exhaustion with NASH and liver fibrosis, further studies are necessary to determine how senescent or exhausted T cells affect HSC activation in vitro and in vivo as part of the progression of metabolic liver disease.

Although the present study has identified a role for insulin resistance-associated alterations in immunophenotype in the pathogenesis of NASH and liver fibrosis, it had several limitations. Liver biopsy is regarded as the gold-standard method of assessing liver fibrosis [36], but we only evaluated liver fat and fibrosis using MR-PDFF and MR elastography in the present study. However, the nature of a population-based study makes liver biopsy impractical, and histology alone is insufficient to confirm a diagnosis of NASH and liver fibrosis in patients with T2D. Although liver biopsy is the reference method for the diagnosis and staging of liver fibrosis, it does have limitations, including sampling error, interobserver variability, and its invasiveness [37, 38]. In addition, the accuracy of MR elastography-based methods for the staging of liver fibrosis has been shown to be equivalent to that of liver biopsy [39, 40]. Secondly, the study sample was exclusively South Korean; therefore, we cannot be certain that the results are applicable to other populations. Thirdly, various confounding factors could not be considered in the multivariate analyses because of the relatively small sample size. Fourthly, this cross-sectional study is valuable in providing a snapshot of the prevalence and association of variables at a specific point in time, we cannot establish a cause-and-effect relationship. However, we observed in this study that the frequencies of senescent T cells increased with the duration of T2D. Although we showed in the previous work, adoptive transfer of CD8 + CD44 + CD153 + T cells also led to a significant deterioration in insulin sensitivity in mice [10], we found no significant correlation between T-cell senescence and duration of T2D in this study (Supplementary Fig. 12a, b). Despite this inevitable limitation of cross-sectional study, we believe that our study can provide the important background for the role of senescent T cells in abnormal glucose homeostasis and progression of liver diseases. However, further studies warrant whether T-cell senescence can influence the progression of liver diseases in humans and mouse models. Finally, we did not consider physical activity in the analysis, which might have affected the results. Therefore, further studies are warranted to better understand the role of T-cell senescence and exhaustion in the progression of metabolic liver disease in patients with T2D and animal models of insulin resistance.

In summary, using flow cytometric analysis of PBMCs and the single-cell transcriptomic analysis of liver-resident cells, we have demonstrated that T-cell senescence and exhaustion in patients with T2D are associated with the progression of fatty liver disease. Furthermore, we have shown that immunophenotyping permits the better identification of individuals with T2D who also have NASH or liver fibrosis, and that it can shed light on the effect of insulin resistance on the progression of metabolic liver disease.

### Supplementary information

Supplementary Information

Uncropped images

## Data Availability

The datasets generated and/or analyzed during the present study are available from the corresponding author on reasonable request.
