# Supplementary material for: Hepatic T-cell senescence and exhaustion are implicated in the progression of fatty liver disease in patients with type 2 diabetes and mouse model with nonalcoholic steatohepatitis
Source: Cell Death Dis. 2023 Sep 21;14(9):618. doi: 10.1038/s41419-023-06146-8 (PMC10514041; doi:10.1038/s41419-023-06146-8)
Supplement: Supplementary file 1 — Supplementary Information [file 41419_2023_6146_MOESM1_ESM.docx]

***Original article***

**Hepatic T-cell senescence and exhaustion are implicated in the progression of fatty liver disease in patients with type 2 diabetes and mouse model with nonalcoholic steatohepatitis**

- Byeong Chang Sim^1,2,#^, Yea Eun Kang^3,#^, Sun Kyoung You^4,#^, Seong Eun Lee^3^, Ha Thi Nga^1,2^, Ho Yeop Lee^1,2^, Thi Linh Nguyen^1,2^, Ji Sun Moon^1^, Jingwen Tian^1,2^, Hyo Ju Jang^1,2^, Jeong Eun Lee^5,^*, Hyon-Seung Yi^1,2,3,^*
- ^1^Laboratory of Endocrinology and Immune System, Chungnam National University School of Medicine, Daejeon, Republic of Korea

^2^Department of Medical Science, Chungnam National University School of Medicine, Daejeon, Republic of Korea

^3^Department of Internal Medicine, Chungnam National University School of Medicine, Daejeon, Republic of Korea

^4^Department of Radiology, Chungnam National University Hospital, Daejeon, Republic of Korea

- ^#^These authors contributed equally to this work.

**Running title:** T-cell senescence in fatty liver disease

**Word count*:*** 3,942 (INTRODUCTION, RESULTS and DISCUSSION)

**Funding**

This work was supported by the Basic Science Research Program, through the National Research Foundation of Korea (NRF), funded by the Ministry of Science, ICT, and Future Planning, Korea (NRF-2021R1A2C4001829). H.S.Y. was supported by the Chungnam National University Hospital Research Fund (2020) and by a grant from the Korea Health Technology R&D Project, through the Korea Health Industry Development Institute (KHIDI), funded by the Ministry of Health & Welfare, Republic of Korea (grant number: HR22C1734).

**Competing interests**

The authors declare no competing interests.

**Correspondence to:**

Hyon-Seung Yi (jmpbooks@cnu.ac.kr) or Jeong Eun Lee (antbantb@naver.com)

**Supplementary Materials and Methods**

***Human liver samples***

Liver samples were collected from 30 participants who underwent lobectomy or segmentectomy at Chungnam National University Hospital because of hepatocellular carcinoma or metastatic liver cancer. The clinical characteristics of the participants are listed in ESM Table 2. Volumes that were not affected by tumor were isolated and used for FACS analysis and real-time PCR. Prior to their inclusion in the study, written informed consent was obtained from all the participants. This protocol and study were approved by the Institutional Review Board of Chungnam National University Hospital (CNUH 2019-11-043).

***Real-time PCR***

Real-time PCR analysis was performed using the primers listed in ESM Table 3. RNA was extracted from human hepatic mononuclear cells using TRIzol Reagent (Invitrogen, Waltham, MA, USA), according to the manufacturer’s instructions. cDNA was synthesized from the same quantity of RNA for each sample using M-MLV reverse transcriptase and oligo-dT primers (Invitrogen), according to the manufacturer’s protocol. Real-time PCR was performed using QuantiTect SYBR Green PCR Master Mix (Qiagen, Hilden, Germany) and an ABI Prism 7000 Sequence Detection System (Applied Biosystems, Foster City, CA, USA). The comparative Ct method was used to quantify transcript expression, which was normalized to that of *18s*. The results were analyzed using the 2^−ΔΔCt^ method, and values are expressed as fold differences from control.

***Generation of mouse model with non-alcoholic steatohepatitis***

Using 8-week-old C57BL/6J male mice, we successfully established animal models of non-alcoholic steatohepatitis (NASH) by administering an Amylin Liver NASH (AMLN) diet composed of 40% high-fat content, 22% high-fructose content, approximately 18% trans-fatty acids, and 2% high-cholesterol for a duration of 30 weeks at the Chungnam National University Hospital (CNUH) Preclinical Research Center and fed a normal chow diet. Subsequently, hepatic mononuclear cells were isolated from two groups of mice: five mice fed a normal chow diet and five mice fed the AMLN diet. These isolated cells underwent comprehensive analysis through flow cytometry and the fluorochrome-conjugated antibodies used were anti-mouse-CD4-PerCP-eFluor710 (46-0041-82, eBioscience), anti-mouse-CD8-AF700 (100730, BioLegend), and anti-mouse-CD279-SB702 (67-9985-82, eBioscience). Single-cell transcriptomics data analysis were conducted to investigate the effects of the dietary intervention on hepatic cellular responses. All animal experiments were approved by the Institutional Animal Care and Use Committee (IACUC) of CNUH (CNUH-021-A0057) and were conducted in accordance with the guidelines of IACUC of CNUH.

***Sirius red staining***

Sections of the left and medial lobes of the liver were fixed with 10% neutral buffered formalin (BBC Biochemical, Mt. Vernon, WA, USA). Five-μm sections of paraffin embedded tissue blocks were stained with 0.1% Sirius Red (Sigma-Aldrich) to examine collagen deposition. For measuring of collagen deposition, 6 areas of each section were taken and analyzed a laser scanning confocal microscope using (FV1000, Olympus Corp., Tokyo, Japan).

***Western blot analysis***

Mouse tissues were homogenized using a TissueLyser II in lysis buffer (50 mM Tris-HCl, pH 7.4; 150 mM NaCl; 1 mM EDTA, pH 8.0; 0.1% Triton X-100) containing a protease inhibitor cocktail (#11836145001, Roche, Basel, Switzerland) and phosphatase inhibitors (04906837001, Roche) on ice for 30 min. After centrifugation at 16,000 g for 15 min, the protein concentrations of the supernatants were measured using a BCA protein assay (#23227, Thermo Fisher Scientific). Fifty micrograms of protein per sample were loaded onto 8–12% polyacrylamide gels and electrophoresis was performed. The separated proteins were then electrotransferred to 0.45 μm PVDF membranes (#IPVH00010, Millipore) at 200 mA for 2 h. Membranes were blocked with 5% skimmed milk (#T145.2, Roth) in TBS/T buffer (20 mM Tris, 150 mM NaCl, 0.1% Tween 20, pH 7.6) for 1 h and then incubated with anti-α-smooth muscle actin antibodies (#ab5694, Abcam, Cambridge, UK) overnight at 4°C. After washing three times with TBS/T, the membranes were incubated with secondary antibodies for 1 h at room temperature and then visualized using ECL solution (#34580, Thermo Fisher Scientific). Target protein levels were normalized to those of glyceraldehyde 3-phosphate (#MA5-15738, Thermo Fisher Scientific, Waltham, MA, USA).

***Subclustering analysis***

To identify T-cell subpopulations, all the cells were subjected to subclustering analysis using Seurat. PCA was performed using differentially expressed genes for dimensionality reduction using the Seurat function RunPCA. The first 13 PCs were used in FindClusters with a clustering resolution of 0.2 to create three initial clusters. Most of the parameters we tried produced similar UMAP clustering, but the use of 13 PCs was associated with the best separation between different cell types. For each cell type, a marker gene was identified using the Seurat function FindAllMarkers and MAST. The subpopulations were visualized using UMAP and the top 13 PCs [1].

***Pseudotime trajectory analysis***

To identify the associated biological processes, such as the interconversion and evolutionary trajectories of the various groups, we used Monocle2. The NewCellDataSet function was used to create a new object for the monocle from the transcript count data for the included cell populations. The results generated from the estimateSizeFactors and estimateDispersions functions assisted us with normalization to account for differences in the amounts of mRNA recovered from the cells and to subsequently perform differential expression analysis. Signature genes expressed in at least 10% of cells in the dataset and with *p* < 0.05 calculated using the differentialGeneTest function were used to define the trajectory. The ReduceDimension function was used to reduce the space down to two dimensions, and the orderCells function was used to order the cells according to their gene expression. Pseudotime-dependent genes were identified using differentialGeneTest and the “fullModelFormulaStr” option “~sm.ns(Pseudotime)”, and smooth expression curves were generated using the plot_pseudotime_heatmap function [2].

***Enrichment analysis***

Pseudotime-dependent genes were further subjected to Gene Ontology (GO) enrichment analysis using the clusterProfiler package (Version 4.4.4) and default settings. All the gene sets in the subontologies were used in the enrichGO function. Enrichment factors were calculated using the counts and BgRatio, which were generated using the enrichGO function [3].

**References**

1. Lee JS, Park S, Jeong HW, Ahn JY, Choi SJ, Lee H, et al. Immunophenotyping of COVID-19 and influenza highlights the role of type I interferons in development of severe COVID-19. Sci Immunol. 2020;5.

2. Qiu X, Hill A, Packer J, Lin D, Ma YA, Trapnell C. Single-cell mRNA quantification and differential analysis with Census. Nat Methods. 2017;14:309-15.

3. Luo H, Xia X, Huang LB, An H, Cao M, Kim GD, et al. Pan-cancer single-cell analysis reveals the heterogeneity and plasticity of cancer-associated fibroblasts in the tumor microenvironment. Nat Commun. 2022;13:6619.

Supplementary Table 1. Characteristics of the participants

| Characteristic | Patients (n = 59) |
| --- | --- |
| Age (years) | 58.7 ± 11.0 |
| Sex | 31 Female (52.5%)  28 Male (47.5%) |
| Body mass index (kg/m^2^) | 26.1 ± 2.9 |
| Type of treatment modalities |  |
| Lifestyle modification | 10 (16.9%) |
| Anti-diabetic drug | 41 (69.5%) |
| Insulin | 1 (1.7%) |
| Drug + insulin | 7 (11.9%) |
| T2D duration (month) | 35.3 ± 37.5 |
| Laboratory findings |  |
| HbA1c (%) | 7.1 ± 1.5 |
| Fasting insulin (uIU/L) | 10.7 ± 9.7 |
| Fasting c-peptide (ng/dL) | 2.1 ± 1.1 |
| Fasting glucose (mg/dL) | 146.1 ± 44.6 |
| hsCRP | 3.6 ± 10.8 |
| AST (U/L) | 25.9 ± 10.0 |
| ALT (U/L) | 27.4 ± 16.7 |
| Total bilirubin (mg/dL) | 0.9 ± 0.4 |
| HOMA-IR | 3.96 ± 4.28 |
| HOMA-β | 54.5 ± 45.7 |
| Triglycerides (mg/dL) | 174.2 ± 96.7 |
| Total Cholesterol (mg/dL) | 160.2 ± 38.2 |
| HDL | 50.7 ± 14.5 |
| LDL | 93.9 ± 38.6 |
| WBC | 7282.2 ± 2340.4 |
| Hemoglobin | 14.1 ± 1.4 |
| Platelet | 243.3 ± 58.4 |
| Albumin | 4.2 ± 0.3 |
| PDFF (%) | 10.2 ± 7.2 |
| Mean liver stiffness (kPa) | 2.0 ± 0.4 |
| Median ATI values (dB/cm/MHz) | 0.72 ± 0.12 |
| 2D-shear-wave elastography (kPa) | 6.86 ± 1.60 |
| NLFS | 1.72 ± 1.03 |
| HIS | 37.56 ± 4.74 |
| NFS | 1.72 ± 1.03 |
| FIB-4 | 1.31 ± 0.55 |

hsCRP, high-sensitivity C-reactive protein; AST, aspartate aminotransferase; ALT, alanine aminotransferase; HOMA-IR, homeostatic model assessment-insulin resistance; HOMA-β, homeostatic model assessment-β-cell function; PDFF, proton density fat fraction; NLFS, nonalcoholic fatty liver disease liver fat score; HIS, hepatic steatosis index; NFS, nonalcoholic fatty liver disease fibrosis score; FIB-4, fibrosis-4 index.

Supplementary Table 2. Metabolic characteristics of the participants, categorized according to HOMA-IR or the presence or absence of obesity

| Variables | HOMA-IR <2.5  (N = 28) | HOMA-IR ≥2.5  (N = 31) | *P*-value | BMI <25 kg/m^2^  (N = 22) | BMI ≥25 kg/m^2^  (N = 37) | *P*-value |
| --- | --- | --- | --- | --- | --- | --- |
| Age (years) | 60.3 ± 8.4 | 57.3 ± 12.8 | 0.283 | 61.0 ± 9.7 | 57.3 ± 11.5 | 0.215 |
| BMI (kg/m^2^) | 25.4 ± 3.0 | 26.8 ± 2.7 | 0.066 | 23.3 ± 1.4 | 27.8 ± 2.2 | <0.001* |
| HbA1c (%) | 6.9 ± 1.4 | 7.2 ± 1.6 | 0.516 | 6.9 ± 1.4 | 7.2 ± 1.5 | 0.591 |
| Fasting insulin (pmol/L) | 5.0 ± 1.7 | 15.8 ± 10.9 | <0.001* | 7.9 ± 5.1 | 12.3 ± 11.3 | 0.088 |
| Fasting C-peptide (pmol/L) | 1.6 ± 0.5 | 2.6 ± 1.2 | <0.001* | 1.8 ± 0.7 | 2.3 ± 1.2 | 0.042* |
| Fasting glucose (mmol/L) | 132 ± 31.2 | 158.8 ± 51.1 | 0.017* | 143.5 ± 44.4 | 147.6 ± 45.2 | 0.737 |
| hsCRP (mg/L) | 1.6 ± 3.2 | 3.1 ± 4.1 | 0.132 | 1.3±2.6 | 2.9±4.2 | 0.120 |
| AST (U/L) | 24.0 ± 7.6 | 27.6 ± 11.7 | 0.165 | 25.3 ± 8.6 | 26.3 ± 10.9 | 0.708 |
| ALT (U/L) | 21.5 ± 8.7 | 32.7 ± 20.3 | 0.009* | 21.2 ± 8.8 | 31.1 ± 19.2 | 0.027* |
| Total bilirubin (mg/dL) | 0.9 ± 0.4 | 0.8 ± 0.4 | 0.682 | 0.8 ± 0.4 | 0.9 ± 0.4 | 0.561 |
| HOMA-IR | 1.5 ± 0.4 | 6.1 ± 5.0 | <0.001* | 2.7 ± 1.9 | 4.7 ± 5.1 | 0.038* |
| HOMA-β | 32.3 ± 18.0 | 74.6 ± 53.5 | <0.001* | 43.7 ± 28.9 | 61.0 ± 52.5 | 0.163 |
| Triglyceride (mg/dL) | 157.3 ± 111.2 | 177.1 ± 86.0 | 0.444 | 151.5 ± 107.9 | 177.4 ± 92.5 | 0.333 |
| Total cholesterol (mg/dL) | 163.5 ± 41.2 | 176.3 ± 55.1 | 0.321 | 163.9 ± 47.0 | 174.1 ± 50.4 | 0.445 |
| NLFS | 0.35 ± 0.81 | 1.76 ± 1.77 | <0.001* | 0.09 ± 1.29 | 1.21 ± 1.87 | 0.017* |
| HIS | 35.74 ± 4.02 | 39.10 ± 4.81 | 0.006* | 33.35 ± 2.70 | 40.06 ± 3.83 | <0.001* |
| NFS | 1.88 ± 1.00 | 1.60 ± 1.05 | 0.296 | 1.98 ± 0.97 | 1.57 ± 1.04 | 0.136 |
| FIB-4 | 1.37 ± 0.49 | 1.27 ± 0.60 | 0.492 | 1.53 ± 0.53 | 1.18 ± 0.52 | 0.017* |
| ATI median | 0.69 ± 0.11 | 0.75 ± 0.11 | 0.070 | 0.64 ± 0.09 | 0.76 ± 0.11 | <0.001* |
| PDFF (%) | 9.0 ± 7.2 | 11.2 ± 7.1 | 0.233 | 6.6 ± 4.6 | 12.3 ± 7.6 | 0.003* |
| Mean liver stiffness (kPa) | 1.85 ± 0.28 | 2.13 ± 0.48 | 0.009* | 1.97 ± 0.30 | 2.02 ± 0.48 | 0.706 |
| Naïve CD4+ | 35.0 ±10.4 | 41.8 ± 13.7 | 0.038* | 34.9 ± 12.7 | 40.7 ± 12.2 | 0.084 |
| Memory CD4+ | 60.5 ± 11.3 | 54.3 ± 15.6 | 0.090 | 60.7 ± 13.3 | 55.2 ± 14.1 | 0.150 |
| Naïve CD8+ | 44.5 ± 16.0 | 47.8 ± 18.0 | 0.451 | 45.8 ± 18.2 | 46.5 ± 16.5 | 0.890 |
| Memory CD8+ | 48.2 ± 16.4 | 47.2 ± 16.8 | 0.823 | 47.7 ± 17.5 | 47.7 ± 16.1 | 0.999 |
| CD4+CD28-CD57+ | 5.8 ± 2.5 | 7.9 ± 4.2 | 0.022* | 6.0 ± 2.8 | 7.5 ± 3.9 | 0.111 |
| CD8+CD28-CD57+ | 38.1 ± 10.2 | 49.5 ± 12.6 | <0.001* | 43.0 ± 12.6 | 44.7 ± 13.0 | 0.634 |
| CD4+CD45RO+CD57+ | 4.9 ± 2.8 | 5.5 ± 3.8 | 0.526 | 5.1 ± 3.2 | 5.3 ± 3.5 | 0.838 |
| CD8+CD45RO+CD57+ | 16.0 ± 8.8 | 21.3 ± 8.1 | 0.019* | 17.9 ± 8.9 | 19.3 ± 8.9 | 0.554 |
| CD4+CD57+IFN-γ+ | 3.7 ± 3.4 | 3.0 ± 2.3 | 0.381 | 3.1 ± 2.7 | 3.5 ± 3.0 | 0.614 |
| CD8+CD57+IFN-γ+ | 12.5 ± 9.8 | 10.8 ± 5.2 | 0.408 | 13.1 ± 9.4 | 10.8 ± 6.4 | 0.264 |
| CD4+CD57+Granzyme+ | 4.6 ± 3.6 | 4.5 ± 2.5 | 0.915 | 4.2 ± 2.7 | 4.8 ± 3.3 | 0.451 |
| CD8+CD57+Granzyme+ | 14.5 ± 9.4 | 18.2 ± 9.2 | 0.138 | 16.7 ± 9.9 | 16.3 ± 9.3 | 0.882 |
| CD4+CD45RO+IFN-γ+ | 11.8 ± 6.4 | 10.4± 3.5 | 0.313 | 11.8 ± 6.4 | 10.7 ± 4.2 | 0.407 |
| CD8+CD45RO+IFN-γ+ | 18.7 ± 8.1 | 17.4 ± 7.9 | 0.528 | 17.3 ± 7.6 | 18.5 ± 8.2 | 0.525 |
| CD4+CD45RO+Granzyme+ | 3.3 ± 2.3 | 3.6 ± 2.7 | 0.657 | 3.6 ± 2.8 | 3.3 ± 2.4 | 0.693 |
| CD8+CD45RO+Granzyme+ | 4.9 ± 4.7 | 4.1 ± 2.5 | 0.405 | 4.5 ± 4.7 | 4.5 ± 3.0 | 0.984 |
| CD4+CD45RO+TNF-α+ | 21.3 ± 11.0 | 22.8 ± 11.0 | 0.610 | 24.5 ± 12.6 | 20.6 ± 9.7 | 0.220 |
| CD8+CD45RO+TNF-α+ | 12.6 ± 7.4 | 17.3 ± 8.4 | 0.026* | 14.4 ± 8.3 | 15.5 ± 8.2 | 0.624 |
| CD4+CD45RO+Perforin+ | 2.0 ± 1,9 | 1.9 ± 1.4 | 0.726 | 2.2 ± 2.1 | 1.8 ± 1.2 | 0.507 |
| CD8+CD45RO+Perforin+ | 2.1 ± 3.0 | 2.7 ± 1.8 | 0.395 | 2.8 ± 3.4 | 2.2 ± 1.7 | 0.486 |

BMI, body mass index; hsCRP, high-sensitivity C-reactive protein; AST, aspartate aminotransferase; ALT, alanine aminotransferase; HOMA-IR, homeostasis model assessment-insulin resistance; HOMA-β, homeostasis model assessment, β-cell function; NLFS, nonalcoholic fatty liver disease liver fat score; HIS, hepatic steatosis index; NFS, nonalcoholic fatty liver disease fibrosis score; FIB-4, fibrosis-4 index; ATI, attenuation imaging; PDFF, proton density fat fraction; CD, cluster of differentiation; IFN-γ, interferon gamma; TNF-α, tumor necrosis factor alpha.

Supplementary Table 3. Clinical characteristics of participants with T2D and with or without NASH or LC

|  | Age | Sex | BMI (kg/m^2^) | AST (IU/L) | ALT (IU/L) | Albumin (g/dL) | Total bilirubin (mg/dL) | Platelet count ×10^3^/mm^3^ |
| --- | --- | --- | --- | --- | --- | --- | --- | --- |
| Control | 50 | M | 21.2 | 28 | 32 | 4.3 | 1.6 | 206 |
|  | 47 | F | 27.2 | 36 | 29 | 4 | 1.6 | 340 |
|  | 55 | M | 18.1 | 34 | 38 | 4.2 | 1.5 | 298 |
|  | 51 | F | 23.8 | 38 | 42 | 4.8 | 1.4 | 356 |
|  | 58 | M | 22.8 | 12 | 28 | 4.3 | 1.3 | 456 |
|  | 60 | M | 24.0 | 20 | 46 | 4.2 | 1.0 | 420 |
|  | 48 | F | 23.8 | 22 | 40 | 4.0 | 1.2 | 355 |
|  | 66 | F | 22.2 | 18 | 28 | 4.6 | 1.8 | 380 |
|  | 69 | F | 23.0 | 16 | 35 | 4.1 | 1.6 | 392 |
|  | 64 | M | 21.6 | 28 | 38 | 4.5 | 1.1 | 258 |
| NASH | 48 | M | 25.2 | 42 | 34 | 3.8 | 1.9 | 293 |
|  | 61 | F | 30.8 | 68 | 90 | 4 | 1.3 | 240 |
|  | 60 | M | 22.6 | 54 | 42 | 4.3 | 1.8 | 250 |
|  | 85 | M | 27.1 | 50 | 52 | 3.9 | 2 | 228 |
|  | 53 | M | 28.5 | 62 | 88 | 4.2 | 1.9 | 356 |
|  | 60 | F | 29.0 | 56 | 80 | 4.5 | 1.5 | 402 |
|  | 58 | F | 31.2 | 42 | 95 | 4.0 | 1.4 | 443 |
|  | 55 | M | 32.0 | 60 | 83 | 4.2 | 1.2 | 460 |
|  | 62 | F | 28.6 | 55 | 77 | 4.0 | 1.0 | 290 |
|  | 53 | M | 24.2 | 54 | 60 | 4.2 | 2.1 | 195 |
| LC | 68 | M | 22.7 | 53 | 70 | 3 | 2.6 | 102 |
|  | 68 | M | 21.3 | 66 | 58 | 3.8 | 1.9 | 130 |
|  | 52 | M | 23 | 55 | 48 | 3.1 | 2.4 | 152 |
|  | 49 | M | 21.5 | 55 | 62 | 3.2 | 3.1 | 110 |
|  | 60 | M | 25.6 | 62 | 80 | 4.0 | 1.5 | 168 |
|  | 63 | M | 24.1 | 54 | 84 | 3.3 | 1.3 | 199 |
|  | 66 | F | 23.0 | 66 | 66 | 3.5 | 2.4 | 98 |
|  | 69 | F | 25.8 | 72 | 90 | 3.4 | 2.0 | 250 |
|  | 61 | F | 21.8 | 38 | 46 | 3.6 | 1.6 | 154 |
|  | 56 | F | 22 | 48 | 56 | 3.8 | 2.8 | 122 |

BMI, body mass index; AFP, alpha fetoprotein; AST, aspartate aminotransferase; ALT, alanine aminotransferase.

**SUPPLEMENTARY FIGURE LEGENDS**

**
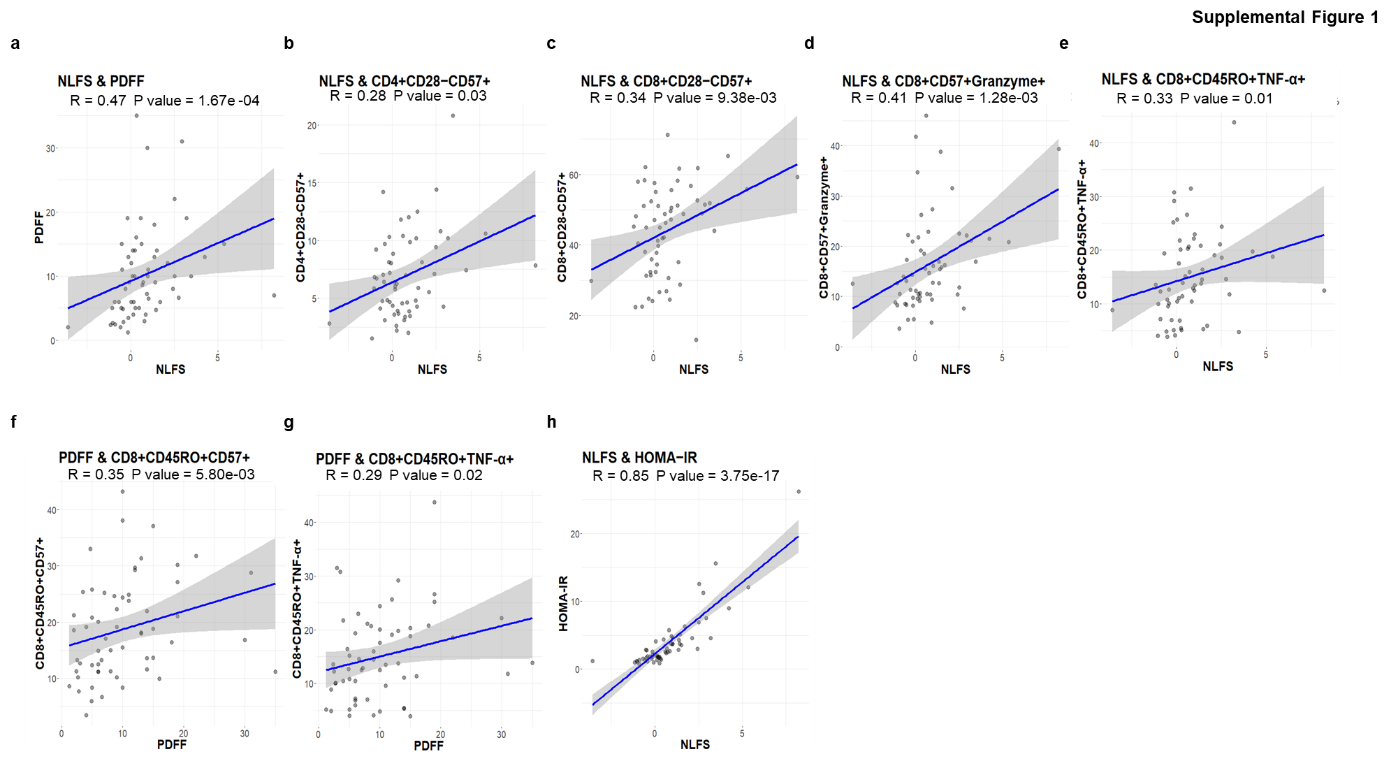
**

**Supplementary Fig. 1. Correlation plots for participants with type 2 diabetes.** *n* = 59. Spearman’s correlation analysis was used. **a** Relationship between PDFF and NLFS. **b, c** Relationships of NLFS with CD4+CD28-CD57+ T cells and CD8+CD28-CD57+ T cells. **d** Relationship between NLFS and CD8+CD57+Granzyme B+ T cells. **e** Relationship between NLFS and CD8+CD45RO+TNF-α+ T cells. **f** Relationship between PDFF and CD8+CD45RO+CD57+ T cells. **g** Relationship between PDFF and CD8+CD45RO+TNF-α+ T cells. **h** Relationship between NLFS and HOMA-IR. NLFS, nonalcoholic fatty liver disease liver fat score; PDFF, proton density fat fraction; TNF, tumor necrosis factor; HOMA-IR, homeostasis model assessment-insulin resistance.


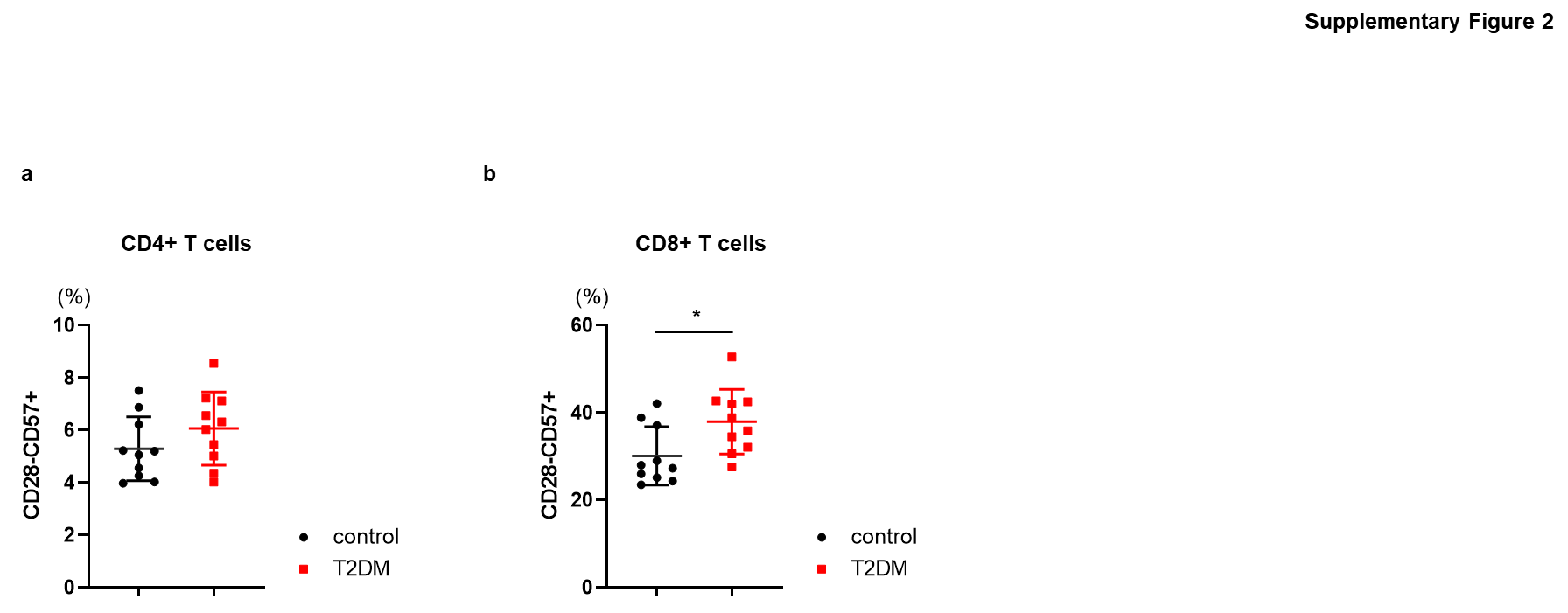


**Supplementary Fig. 2. Immunophenotypic characteristics of peripheral blood mononuclear cells from subjects with normoglycemia and participants with T2D.** a, b Percentages of CD28–CD57+ cells within the CD4+ and CD8+ T-cell population of control subjects and T2D patients. The unpaired *t*-test was used for data analysis. **P* < 0.05. Data are presented as the mean ± SEM. T2D; type 2 diabetes.


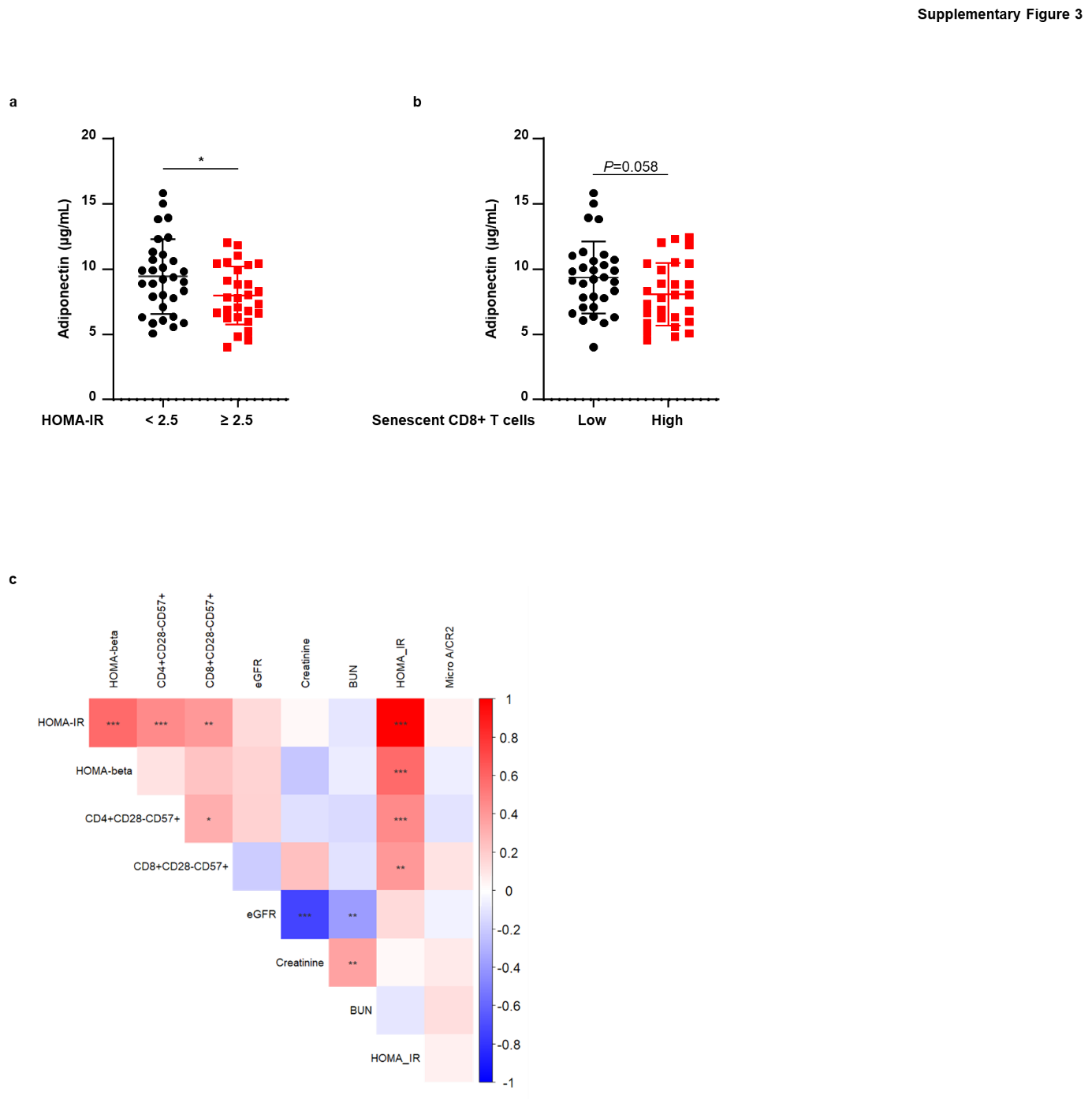


**Supplementary Fig. 3. Serum adiponectin levels in participants with T2D.** **a,b** Serum levels of adiponectin in patients with T2D. Adiponectin levels were analyzed according to HOMA-IR and populations of senescent CD8+ T cells. **c** Correlogram, with the depth of shading representing the magnitude of the correlation and positive and negative correlations being shown in blue and red, respectively. The unpaired *t*-test was used for data analysis. **P* < 0.05. Data are presented as the mean ± SEM. BUN; blood urea nitrogen; eGFR; estimated glomerular filtration rate; HOMA-beta, homeostasis model assessment-β-cell function; HOMA-IR, homeostatic model assessment-insulin resistance.


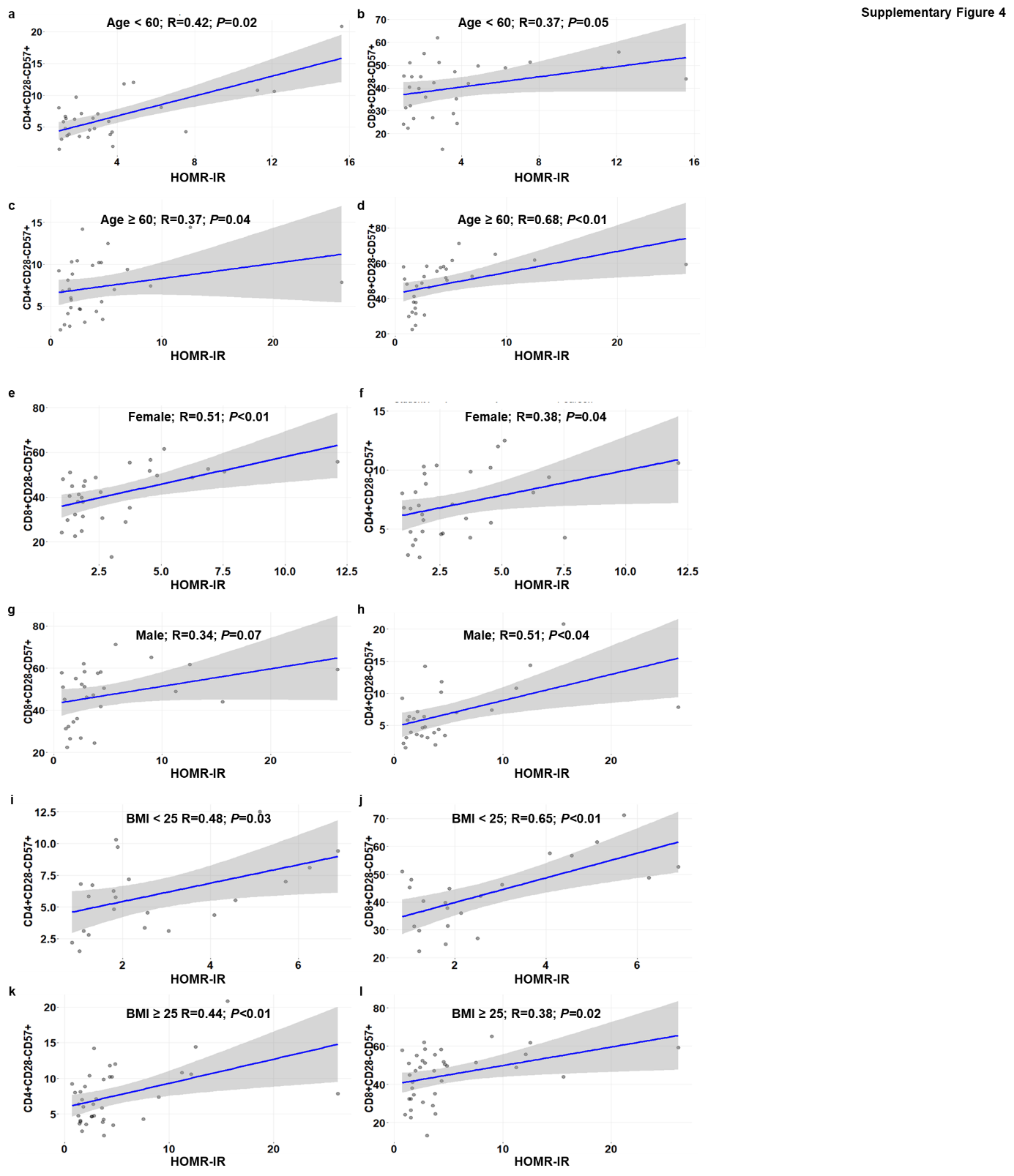


**Supplementary Fig. 4. Correlation plots for participants with type 2 diabetes.** Spearman’s correlation analysis was used. **a–d** Relationship between HOMA-IR and age. **e–h** Relationship between HOMA-IR and gender. **i–l** Relationship between HOMA-IR and BMI. BMI; body mass index; HOMA-IR, homeostatic model assessment-insulin resistance.


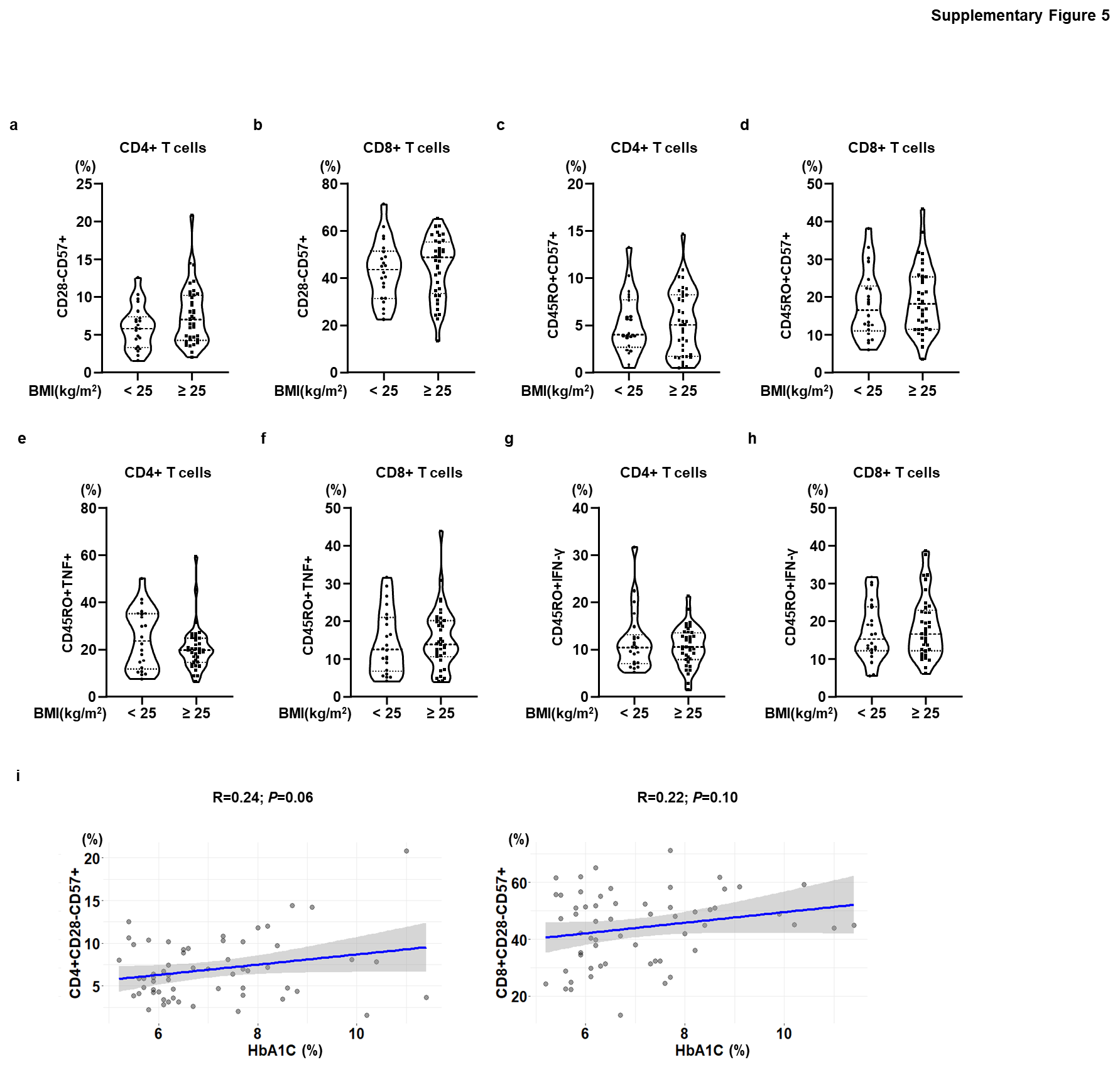


**Supplementary Fig. 5. Immunophenotypic characteristics of peripheral blood mononuclear cells from participants with T2D according to BMI.** **a, b** Percentages of CD28–CD57+ cells and **c, d** percentages of CD45RO+CD57+ cells within the CD4+ and CD8+ T-cell population of participants with T2D. **e, f** Percentages of TNF-producing CD45RO+ cells within the CD4+ and CD8+ T-cell population. **g, h** Percentages of IFN-γ-producing CD45RO+ cells within the CD4+ and CD8+ T-cell population. **i** Relationship between HbA1c and senescent CD4+ or CD8+ T cells. Asterisks indicate significant differences between participants with T2D and BMI <25 kg/m^2^ or ≥25 kg/m^2^. The unpaired *t*-test was used for data analysis. Data are presented as the mean ± SEM. BMI, body mass index; IFN-γ, interferon gamma; TNF, tumor necrosis factor.


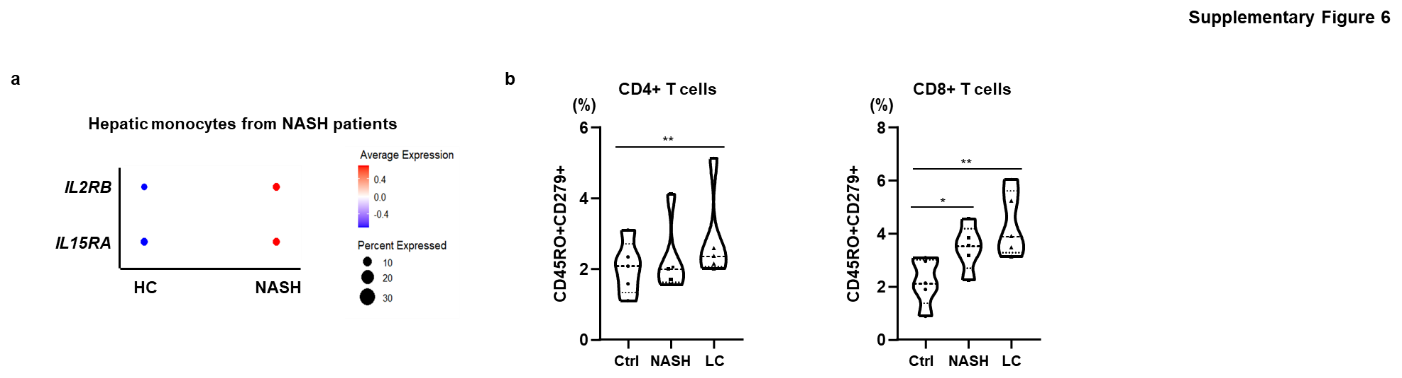


**Supplementary Fig. 6. Immunophenotype of hepatic monocytes and T cells from participants with T2D and with or without NASH or liver cirrhosis.** **a** Expression of *IL2RB* and *IL15RA* in hepatic monocytes, according to single-cell transcriptomic data from the Gene Expression Omnibus (GSE159977). The size of each dot represents the percentage expression of a gene, compared to that of all other transcripts, and the color gradient of the dot indicates the mean expression of the gene. **b** Percentage of CD45RO+CD279+ cells within the CD4+ and CD8+ T-cell population from participants with T2D and with or without NASH or liver cirrhosis. Data are presented as the mean ± SEM. Data in **b** were analyzed using one-way ANOVA. **P* < 0.05, ***P* < 0.01. HC, healthy controls; Ctrl, controls with T2D; NASH, nonalcoholic steatohepatitis; LC, liver cirrhosis; T2D, type 2 diabetes.


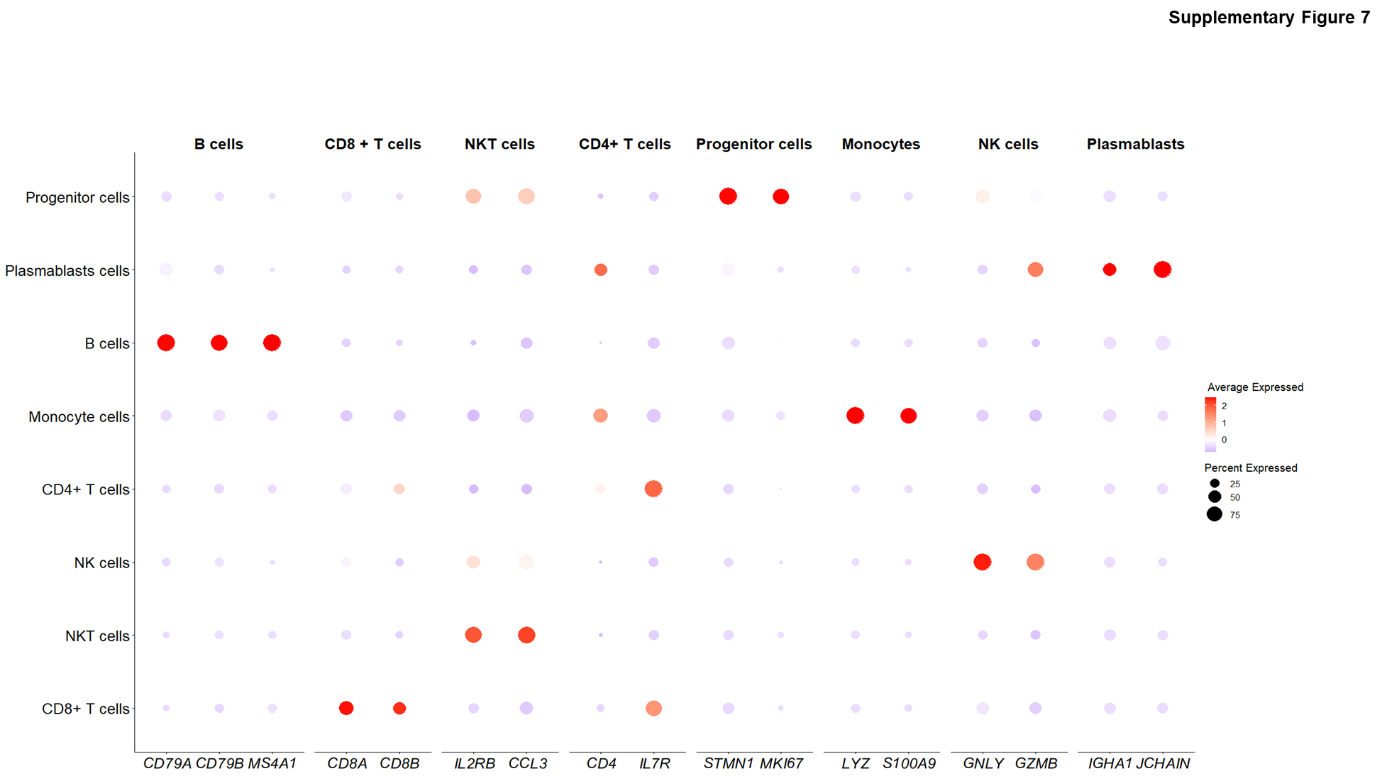


**Supplementary Fig. 7. Expression levels of marker genes for the identification of hotspots of clustering in single hepatic cells from controls and participants with nonalcoholic steatohepatitis.**


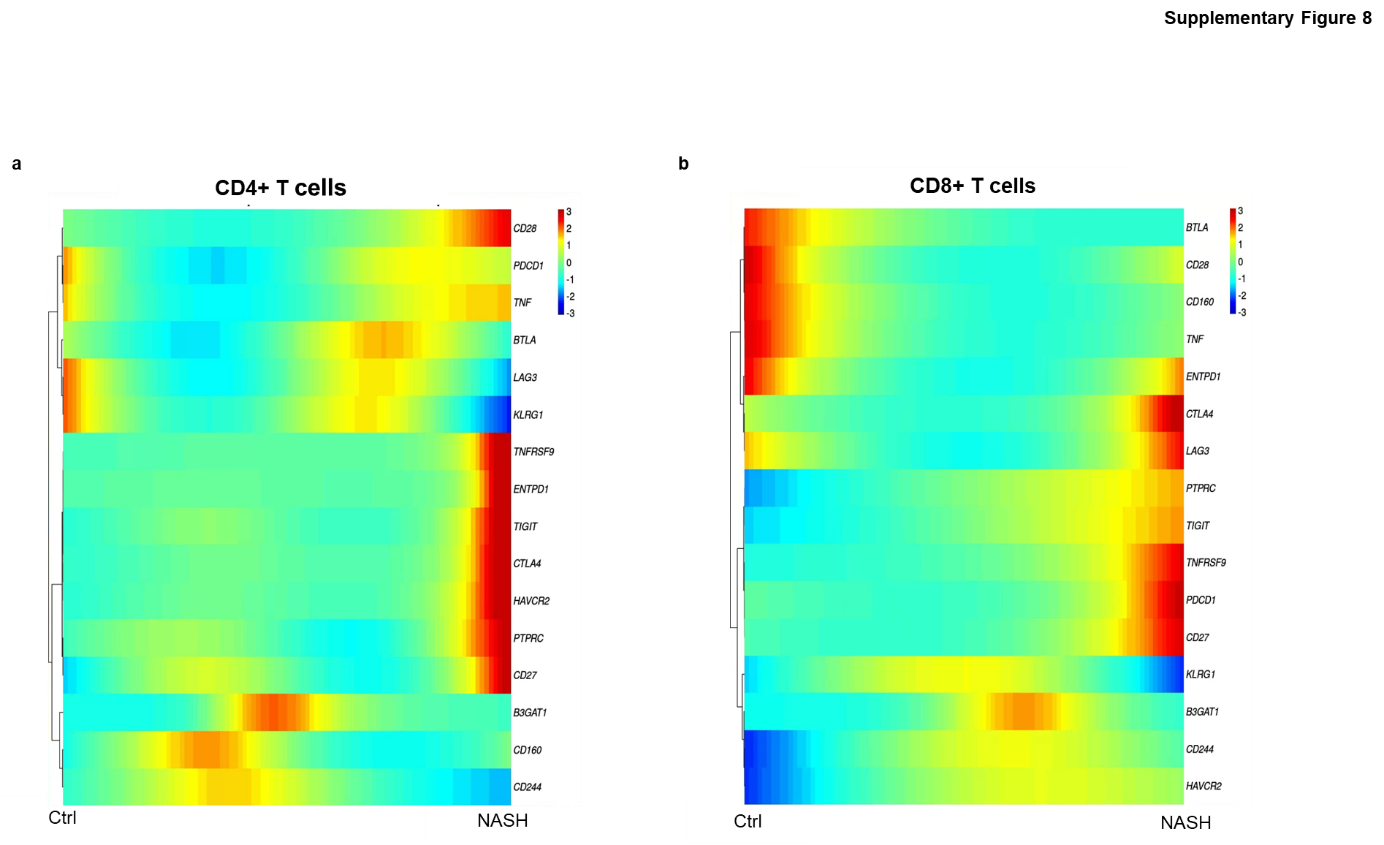


**Supplementary Fig. 8. Trajectory analysis of hepatic T cells from controls and participants with nonalcoholic steatohepatitis.** **a, b** Relative expression patterns of representative genes related to T-cell senescence and exhaustion in the trajectory analysis are plotted along a pseudotime axis. The color indicates the relative gene expression, calculated using Monocle 2.


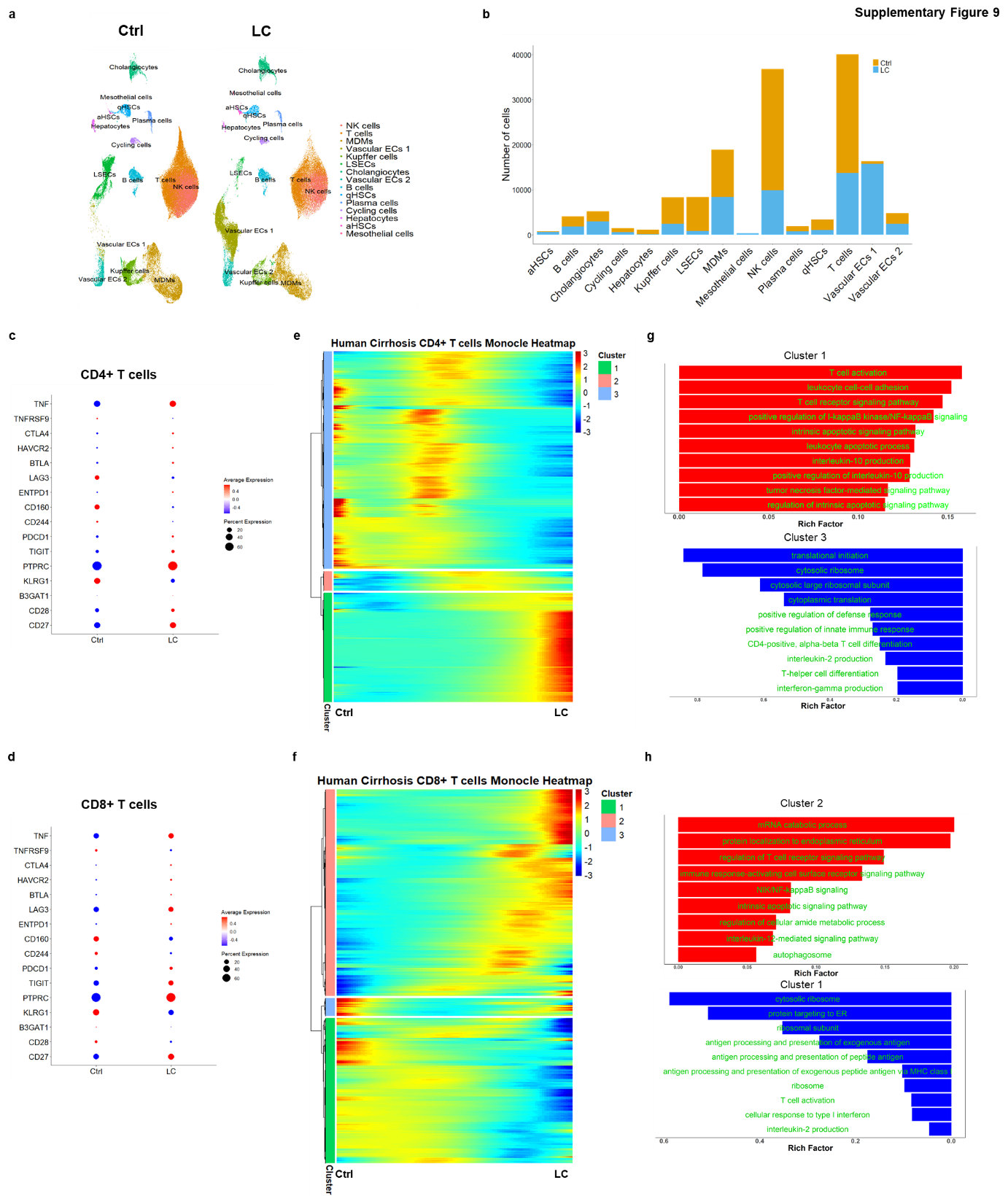


**Supplementary Fig. 9. Single-cell transcriptomic analysis of the livers of participants with liver cirrhosis.** Liver samples were analyzed to better characterize the tissue-infiltrating immune cells. Using the E-MTAB-10553 dataset, 152,113 single cells from the participants with liver cirrhosis (*n* = 10) and healthy controls (*n* = 3) were subjected to single-cell transcriptomic analysis. **a** Single-cell transcriptomic data-based UMAP representation of the FlowSOM-guided clustering of CD45+ cells in participants with or without liver cirrhosis. Colors indicate each annotated cell type. **b** Bar plot representing the relative contribution of hepatic cells from participants with or without liver cirrhosis. **c, d** Dot plots displaying the senescence and exhaustion marker genes for the CD4+ and CD8+ T cells identified. The size of the dot represents the proportion of the cell population that expresses each gene, and the color indicates the level of expression. **e, f** Gene expression of hepatic CD4+ and CD8+ T cells from participants with liver cirrhosis and healthy controls, analyzed along a latent time/pseudotime axis corresponding to the pathological progression of each cell. **g, h** Bar plots showing pathway enrichment analysis. Rich factor: ratio of the expression of differentially expressed genes annotated in this pathway to that of all the genes annotated in this pathway. A higher rich factor implies higher expression.


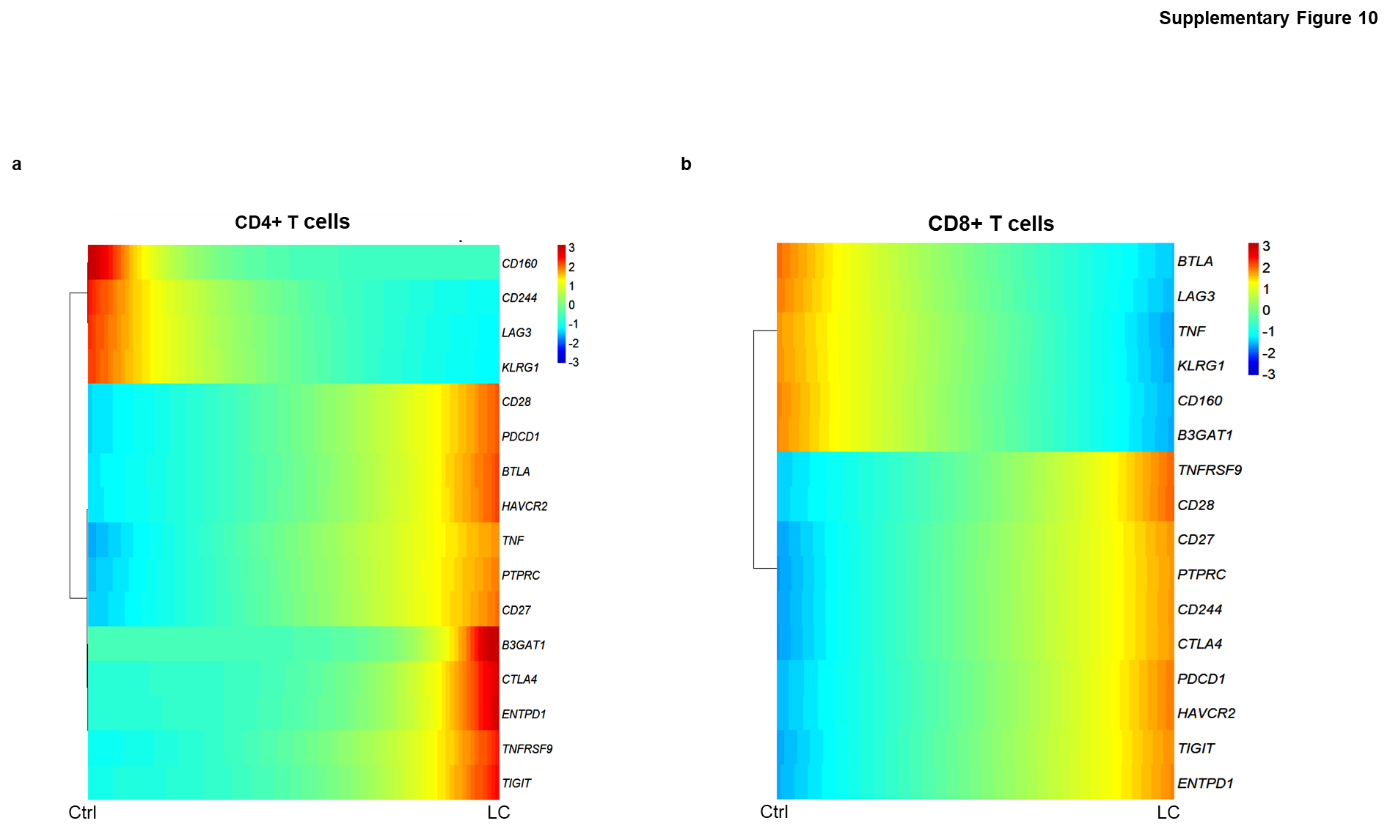


**Supplementary Fig. 10. Results of the trajectory analysis of hepatic T cells from controls and participants with liver cirrhosis.** **a, b** Relative expression patterns of representative genes related to T-cell senescence and exhaustion in the trajectory analysis are plotted along a pseudotime axis. The color indicates the relative gene expression, calculated using Monocle 2.

**
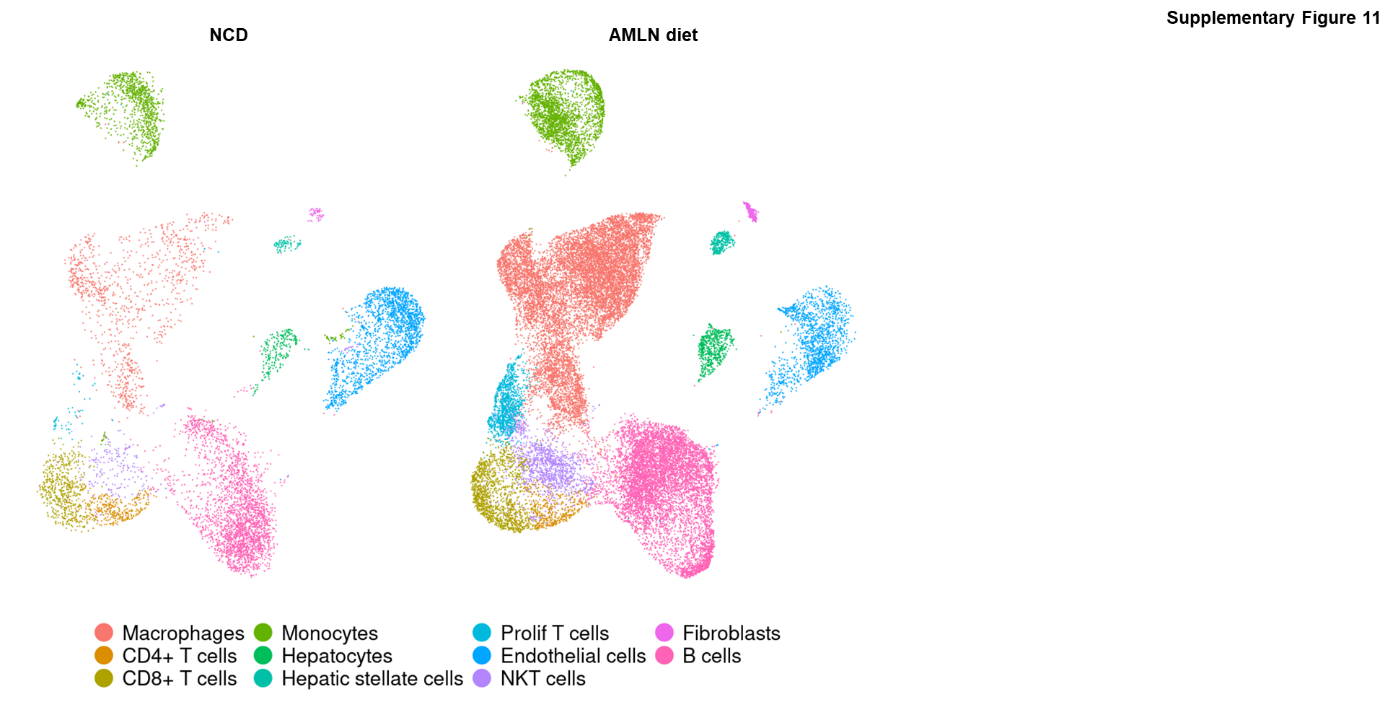
**

**Supplementary Fig. 11. Hepatic single-cell transcriptomic data analysis of mouse modes with NASH.** Approximately 29,996 single cells from mice fed NCD or AMLN diet were subjected to single-cell transcriptomic analysis and analyzed by UMAP representation.

**
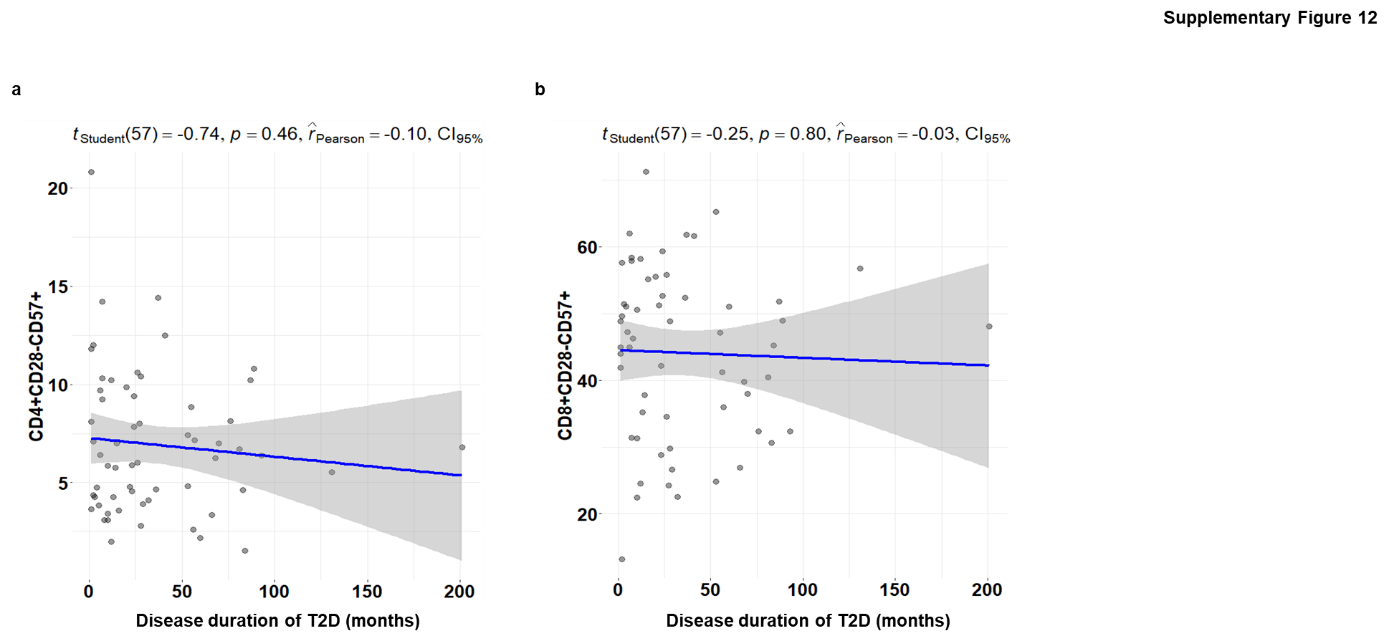
**

**Supplementary Fig. 12. Correlation plots for participants with type 2 diabetes.** Spearman’s correlation analysis was used. **a,b** Relationship between disease duration of type 2 diabetes and senescent CD4+ or CD8+ T cells in participants with type 2 diabetes.
