# Supplementary material for: Hepatic T-cell senescence and exhaustion are implicated in the progression of fatty liver disease in patients with type 2 diabetes and mouse model with nonalcoholic steatohepatitis
Source: Cell Death Dis. 2023 Sep 21;14(9):618. doi: 10.1038/s41419-023-06146-8 (PMC10514041; doi:10.1038/s41419-023-06146-8)
Supplement: Supplementary file 2 — Uncropped images [file 41419_2023_6146_MOESM2_ESM.pptx]

## Slide 1
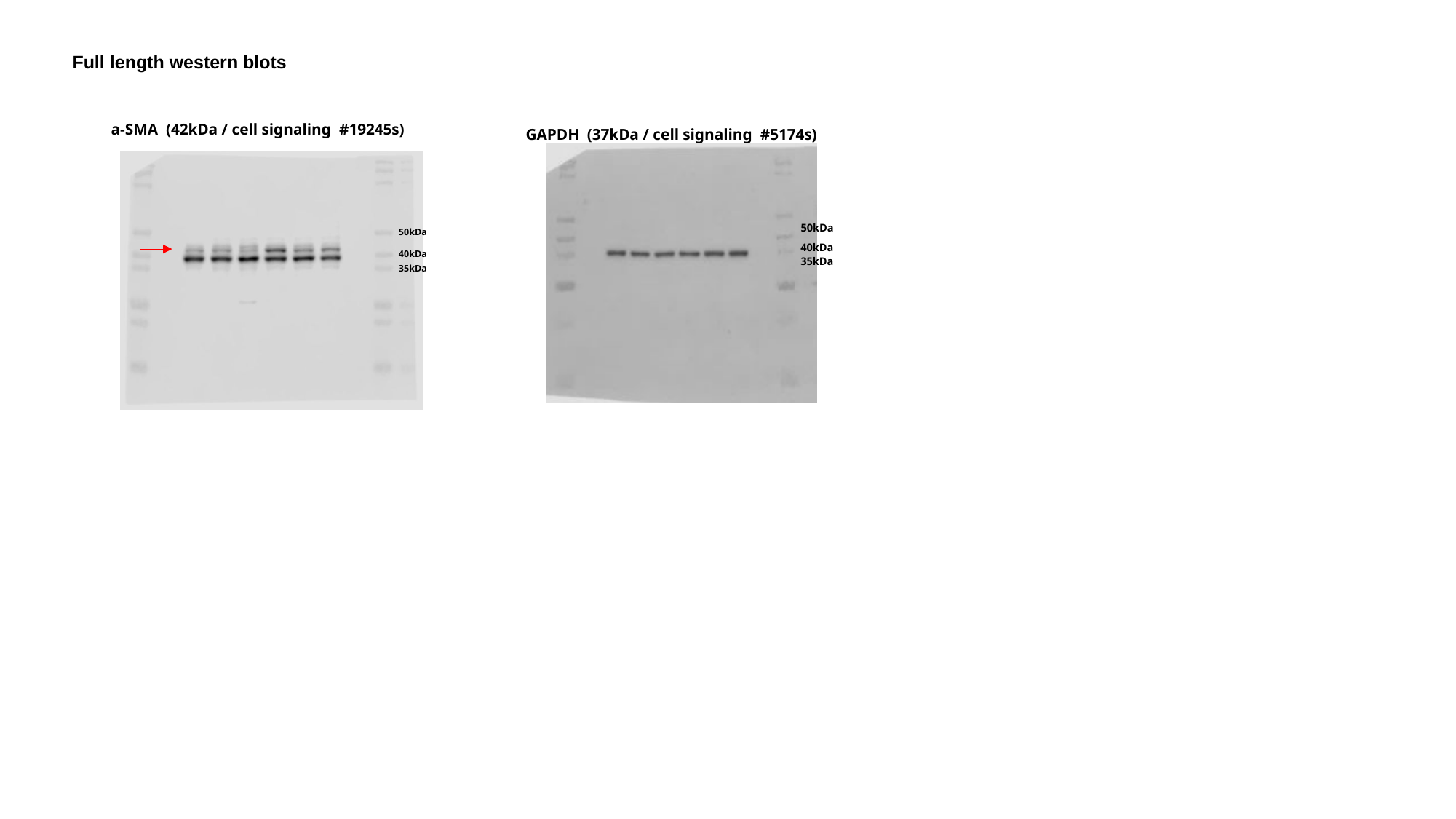

Full length western blots
a-SMA (42kDa / cell signaling #19245s)
GAPDH (37kDa / cell signaling #5174s)
50kDa
50kDa
40kDa
40kDa
35kDa
35kDa
